# Evaluation of Encapsulation Potential of Selected Star-Hyperbranched Polyglycidol Architectures: Predictive Molecular Dynamics Simulations and Experimental Validation

**DOI:** 10.3390/molecules28217308

**Published:** 2023-10-28

**Authors:** Mateusz Gosecki, Malgorzata Urbaniak, Nuno Martinho, Monika Gosecka, Mire Zloh

**Affiliations:** 1 Centre of Molecular and Macromolecular Studies, Polish Academy of Sciences, Sienkiewicza 112, 90-363 Lodz, Poland; mateusz.gosecki@cbmm.lodz.pl (M.G.); malgorzata.urbaniak@cbmm.lodz.pl (M.U.); 2 IBB—Institute for Bioengineering and Biosciences, and Associate Laboratory i4HB—Institute for Health and Bioeconomy at Instituto Superior Técnico, Universidade de Lisboa, Av. Rovisco Pais, 1049-001 Lisboa, Portugal; nunomartinho@outlook.com; 3 UCL School of Pharmacy, University College London, 29/39 Bruunswick Square, London WC1N 1AX, UK; 4 Faculty of Pharmacy, University Business Academy, Trg Mladenaca 5, 21000 Novi Sad, Serbia

**Keywords:** hydrophobic drug, encapsulation, molecular dynamics simulation, clotrimazole, unimolecular micelles, star-hyperbranched copolymer, polyether

## Abstract

Polymers, including non-linear copolymers, have great potential in the development of drug delivery systems with many advantages, but the design requires optimizing polymer–drug interactions. Molecular dynamics (MD) simulations can provide insights into polymer–drug interactions for designing delivery systems, but mimicking formulation processes such as drying is often not included in in silico studies. This study demonstrates an MD approach to model drying of systems comprising either hydrophilic tinidazole or hydrophobic clotrimazole drugs with amphiphilic hyperbranched copolyethers. The simulated drying protocol was critical for elucidating drug encapsulation and binding mechanisms. Experimentally, two polymers were synthesized and shown to encapsulate clotrimazole with up to 83% efficiency, guided by interactions with the hydrophobic core observed in simulations. In contrast, tinidazole is associated with surface regions, indicating capacity differences between drug types. Overall, this work highlights MD simulation of the drying process as an important tool for predicting drug–polymer complex behaviour. The modelled formulation protocol enabled high encapsulation efficiency and opened possibilities for the design of delivery systems based on computationally derived binding mechanisms. This demonstrates a computational–experimental approach where simulated drying was integral to elucidating interactions and developing optimized complexes, emphasizing the value of molecular modelling for the development of drug delivery formulations.

## 1. Introduction

Many hydrophobic drugs exhibit valuable therapeutic properties; however, their low solubility in water limits their bioavailability. Many efforts are focused on increasing the solubility of hydrophobic drugs in the aqueous medium. Micelles constructed of amphiphilic di- or tri-block copolymers are routinely applied as carriers of hydrophobic active substances. The fundamental limitation of micelles as drug carriers, however, is their low stability upon environmental changes. The disintegration of micelles due to the dilution in the physiological conditions, the shear force [1], or other factors including protein binding can result in a burst release of encapsulated drugs.

In contrast to the concentration-dependent limited stability of classical micelles, unimolecular micelles, i.e., single-molecular amphiphilic core-shell constructs, are intrinsically stable and thus preserve their structure regardless of the concentration. Unimolecular micelles can be constructed of star-shaped [2] dendrimer-type [3,4,5] or hyperbranched polymers [6,7]. Recently, the interest in unimolecular micelles, which enhance the solubilization of hydrophobic drugs, is increasing [8,9]. Amongst hyperbranched polymers, hyperbranched polyglycidol (HbPGL), due to its biocompatibility [6,7,10,11,12,13,14], low cytotoxicity, low viscosity [15], high hydrophilicity [6], and numerous functional groups [15], deserves a particular interest as a prospective drug carrier. A tree-like structure of HbPGL delivers nanosized pockets for low-molecular drug molecules [7,16,17]. The synthesis of unimolecular micelles based on hyperbranched polyglycidol requires the selective hydrophobization of its core. This can be achieved by the modification of monohydroxyl moieties incorporating hydrophobic groups. The hydrophobization of monohydroxylated constitutional units of hyperbranched polyglycidol, however, significantly changes the rheological properties of formed hydrogels. The hydrophobization of HbPGL’s core gives the possibility of water-insoluble drug solubilization within HbPGL. The structure of hydrophobic units incorporated into the HbPGL core, however, has to be carefully tailored to maintain the solubility of macromolecules in water and assure the solubility of the drug. Recently, we demonstrated that the drug-loading capacity of the internally hydrophobized HbPGL was strictly dependent not only on the degree of hydrophobization of monohydroxylated units present in the core but also on the type of bond applied for the covalent immobilization of hydrophobic groups, i.e., benzoyl ester or phenyl carbamate linkages [9]. Estimating the optimal amphiphilic construct for the encapsulation of clotrimazole, a highly hydrophobic drug, required the synthesis of numerous constructs differing in the degree of the core hydrophobization, which was both time- and material-consuming.

The use of computational chemistry approaches can aid efficient screening and design of hyperbranched polymers for drug encapsulation, optimizing their properties and achieving better drug delivery performance [18]. Particularly, molecular dynamics simulations can provide a detailed and mechanistic understanding of drug encapsulation and interactions with hyperbranched polymers [19,20]. This knowledge is invaluable for guiding experimental efforts, accelerating drug delivery system development, and optimizing drug delivery strategies for specific therapeutic applications.

**Figure 1:**
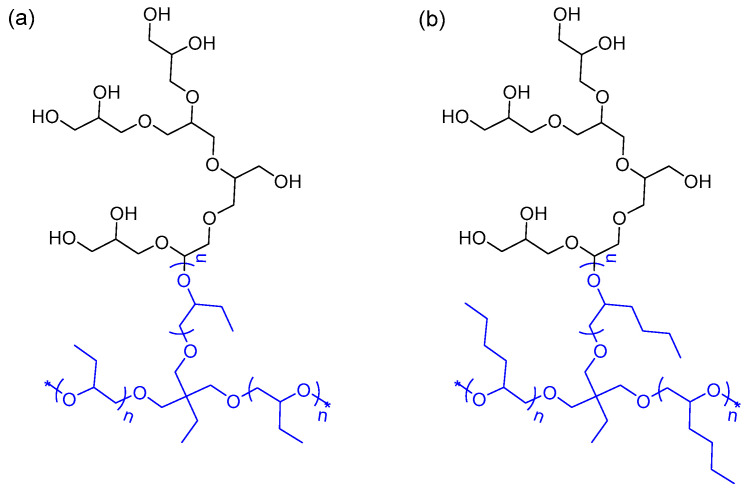
Copolyether-based amphiphilic HbPGL constructs with (**a**) poly(1,2-epoxybutane)-*co*-HbPGL (R14) and (**b**) poly(1,2-epoxyhexane)-*co*-HbPGL (R17) comprising hydrophobic cores and hydrophilic polyglycidol outer layer. The cores of both molecules comprise five repeating units (*n* = 5) and are coloured blue. Both molecules have three HbPGL branches, with only one branch HbPGL shown for simplicity, the asterisk (*) indicates the conjugation points for the remaining branches.

In this article, the potential of two amphiphilic star-hyperbranched HbPGL-based copolyethers (Figure 1) was evaluated for the enhancement of drug solubilization. The effects of n-alkyl length of repeating units in the hydrophobic star-shaped core surrounded by highly hydrophilic corona were studied on the encapsulation of two model drugs using molecular dynamics simulations. Furthermore, the molecular dynamics simulations of drug–polymer mixtures in different solvent systems informed the experimental design in formulation preparations. The presented method can be helpful in the determination of both composition and macromolecules’ architecture suitable for the achievement of enhanced solubilization of a specific drug in the aqueous medium as well as proposed formulation preparation strategies based on the understanding of polymer–drug intermolecular interactions. These studies also provide a strong foundation for the development of computational approaches to establish a relationship between the nature and number of polymers repeating units and their drug loading capacity.

## 2. Results and Discussion

The drug encapsulation studies were conducted for two star-hyperbranched copolyethers (R14 and R17, see Supplementary Materials) and two model drugs with different lipophilic properties (slightly hydrophilic tinidazole and hydrophobic clotrimazole). Initially, in silico studies were conducted to evaluate interactions between R17 and tinidazole, which led to a proposed experimental design for the evaluation of polymer encapsulation of both drugs tinidazole and clotrimazole, for which full in silico and experimental were conducted in parallel.

### 2.1. In Silico Studies

#### 2.1.1. 3D Structure Generation and Surface Properties

Preliminary unpublished experimental results indicated that HbPGL polymers tend to have a range of molecular weights due to some monomers not being incorporated during syntheses resulting in hyperbranched molecules with missing monomers (HbPMM). The synthesis of HbPGL shell by anionic polymerization of glycidol is not a controlled process making the polymer a mixture of different isomers.

The hyperbranched polyglycidol is synthesized via uncontrolled anionic polymerization of glycidol by slow monomer dosing to reduce the formation of cyclic by-products. To explore the effects of such defects on the molecular properties, five different structures of R14 and R17 were generated using in-house developed scripts. These were subjected to a protocol comprising of simulated annealing (SA) and molecular dynamics (MD) simulation of explicitly solvated systems to remove the bias of minimizing the structures using implicit solvation implemented in RDKit.

**Figure 2:**
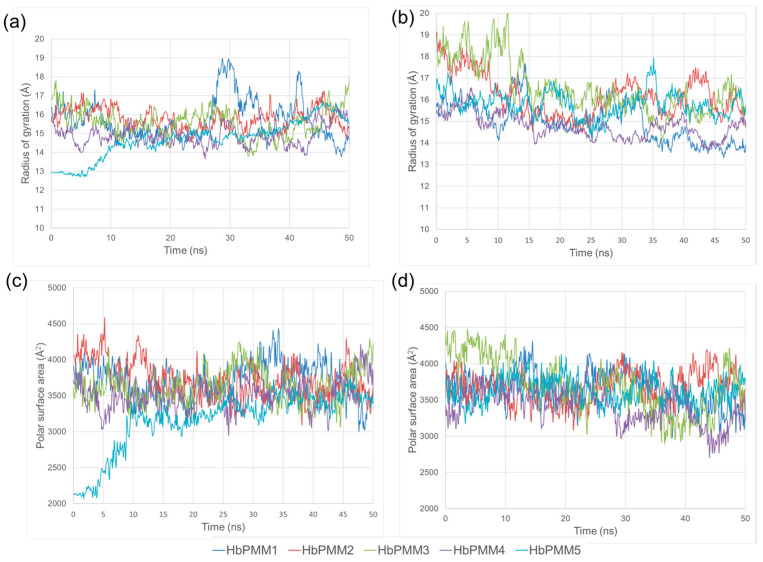
Macroscopic properties for five HbPMM species of R14 and R17 calculated over 50 ns MD simulation at 300 K using Desmond software version 2022.4 and OPLS-2005 force field: the radius gyration is shown in panels (**a**) R14 and (**b**) R17, while the polar surface area is shown in panels (**c**) R14 and (**d**) R17.

The shape and surface properties from the resulting trajectories of the MD production run were compared for each HbPMM molecule via radius gyration (RG) and polar surface area (PSA) (Figure 2). SA protocols include heating the systems to 1000 K, which results in final frame conformations that are significantly different as their RGs can be different by up to 4 Å. However, after 15 ns simulation at 300 K, the systems appear to be equilibrated, with the RGs fluctuating around 15.4 Å and 15.3 Å for R14 and R17, respectively. The fluctuations of the radius of gyrations of both hyperbranched polymers indicated that molecules are flexible and that their conformations change continuously for each of the five different HbPMMs. It is interesting that R17 appears to be marginally smaller despite having a bulkier monomer in the core, which may be a result of the higher degree of core hydrophobicity. At the same time, the average polar surface area of R14 and R17 is nearly the same, due to the same hydrophilic corona. It should be noted that molecular dynamics simulations of polymers should generally take into account the variation in their size and possible imperfections in their structures, which may arise due to experimental constraints.

**Figure 3:**
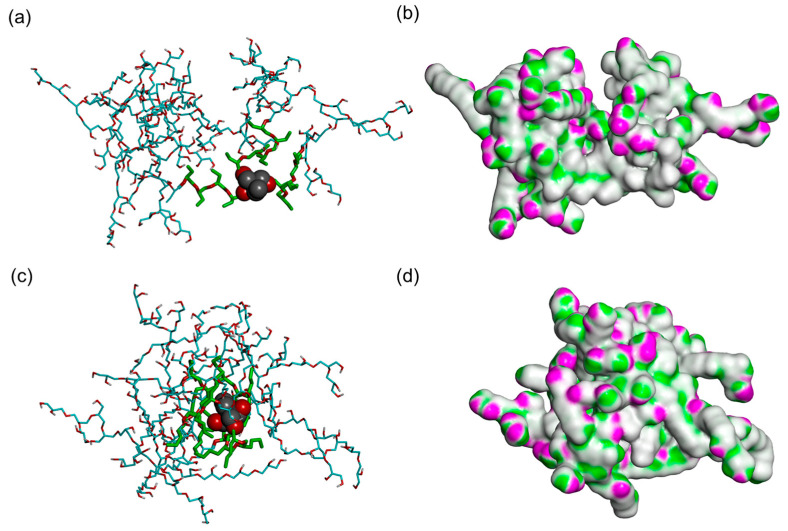
Final frames of the MD simulation trajectories of polymers based on HbPGL shown as a mixture of CPK and stick representation: (**a**) R14 and (**c**) R17 and surface representation coloured according to hydrogen bonding potential: (**b**) R14 and (**d**) R17. The core of the polymers is shown in CPK representation, hydrophobic polyether residues are shown as thick sticks coloured in green, and hydrophilic corona residues are shown as thin sticks coloured in cyan; only polar hydrogen atoms are shown for clarity. Purple coloured surface indicates the hydrogen donor potential, and green coloured surface indicates the hydrogen bond acceptor potential.

Overall, the molecular properties of the five HbPMM species for both types are marginally different, and therefore, further encapsulation studies were conducted using arbitrarily selected HbPMM1 molecules of R14 and R17 (Figure 3). The R17 appears to be more compact compared to R14, which is in agreement with the assumption that bulkier hydrophobic residues drive closer interactions between them and allow the hydrophilic layer to encompass the core in the interior. The distribution of hydrogen bonding acceptors and donor groups appears to be distributed similarly on the surface of two hyperbranched polymers.

#### 2.1.2. Molecular Dynamics Simulations of R17 and Tinidazole

**Figure 4:**
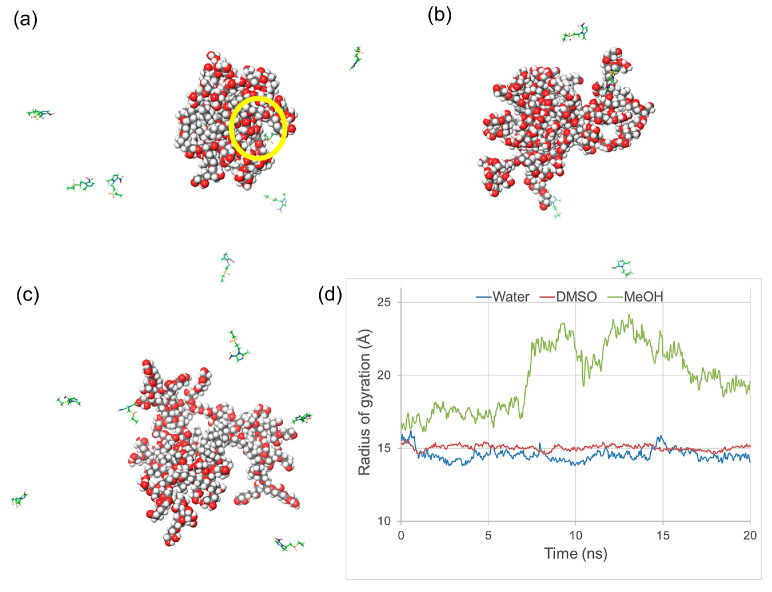
Final frames of the MD simulation trajectories of a mixture of R17 and six molecules of tinidazole in explicit solvent: (**a**) water, (**b**) DMSO, and (**c**) methanol. Polymer is shown in CPK representation, and tinidazole molecules are shown in stick representation with carbon atoms coloured in green. The yellow circle indicates the position of a single tinidazole molecule near the polymer. (**d**) RG values calculated for R17 in each frame of 20 ns MDs simulations in different solvents.

A conformation from the MD simulation of R17 extracted from the final frame of the simulation was used to prepare systems for simulations of mixtures by arranging six tinidazole molecules around the polymer and fully solvating them with either water, DMSO, or methanol. Each system was equilibrated and simulated using a well-established NPT protocol for 20 ns at 300 K. It was found that the polymer was getting further compacted in water, while it was swelling in DMSO and methanol leading to increased RG in non-polar solvents (Figure 4). There is an apparent lack of significant interactions between a model hydrophilic drug and R17 in all solvent systems. Close inspection of the final frames of MD simulations and radius gyration values of the R17 (Figure 4d) indicated that polymer dissolved in methanol may be the best system for further encapsulation of drug molecules due to exposing the hydrophobic core for the formation of interactions. However, as there is a lack of intermolecular interactions between two types of molecules, it was proposed that removing methanol would force drug encapsulation.

**Figure 5:**
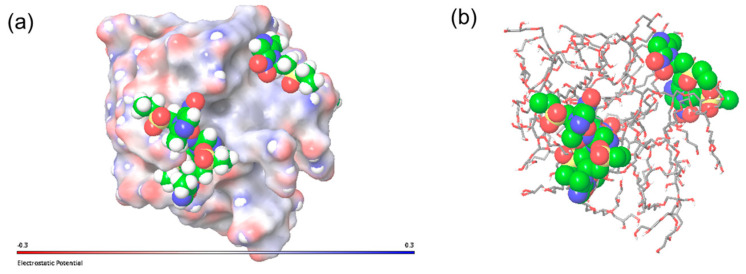
Final frames of the MD simulation trajectories of the mixture of R17 and six molecules of tinidazole at the final stage of methanol removal that simulate the drying process. The tinidazole molecules are shown in CPK representation, and R17 is shown (**a**) as an interpolated charge surface and (**b**) stick representation.

The most currently available software packages use NPT or NVT ensembles for molecular dynamics simulations of various systems but are not suitable for simulations of drying processes. We tested “Model system regeneration” implemented in Desmond software (version 2022.4) that allows the possibility of changing the composition of previously simulated systems and preparing them for further simulations. This allowed us to remove methanol molecules and start new simulations with the regenerated system. Removal of solvent molecules away from a set distance away from the non-volatile molecules could be a crude approximation of a drying process. Further simulations were carried out using NVT ensemble settings. The drying process was mimicked by removing layers of solvents in stages to simulate the gradual changes in the simulated system and allow the formation of polymer–drug complexes without introducing bias. The methanol removal was conducted in six steps as described in Section 3.8. The final frame of the trajectory from the MD simulation of the dried system showed that all six tinidazole molecules were interacting with R17 (Figure 5) and forming on average three hydrogen bonds between them. The drying process and formation of drug–polymer complexes can be visualized via a set of movies given as Supplementary Materials.

**Figure 6:**
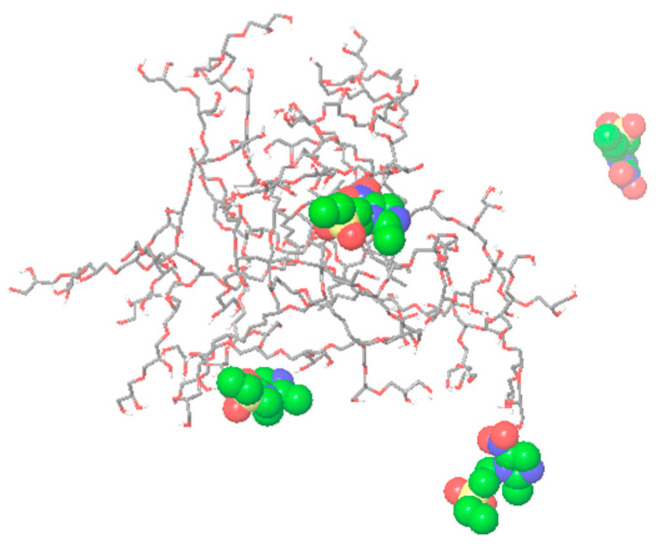
The final frame of the 50 ns MD simulation trajectory of the mixture of R17 and six molecules of tinidazole in water. The starting point for the simulation was the system obtained from the drying process, namely the simulation of the mixture after the gradual removal of methanol.

These results indicated that the R17 folded back to a compact structure in the absence of methanol, while the tinidazole molecules did not interact with the hydrophobic core. This complex was fully solvated in water, and the new molecular system was subjected to MD simulation to explore how tinidazole would redissolve. It was observed that after the end of the 50 ns MD simulation, two out of six drug molecules were still attached to the R17 (Figure 6). Since the first drug molecule was released into solution only after the 5 ns of simulation, it suggests that R17 has the potential to be used as a slow-release formulation of tinidazole, and importantly, this in silico approach may inform formulation. Therefore, further MD simulation of mixtures of hyperbranched polymers and hydrophobic drugs such as clotrimazole should be carried out.

#### 2.1.3. Molecular Dynamics Simulations of Clotrimazole Polymer Mixtures

**Figure 7:**
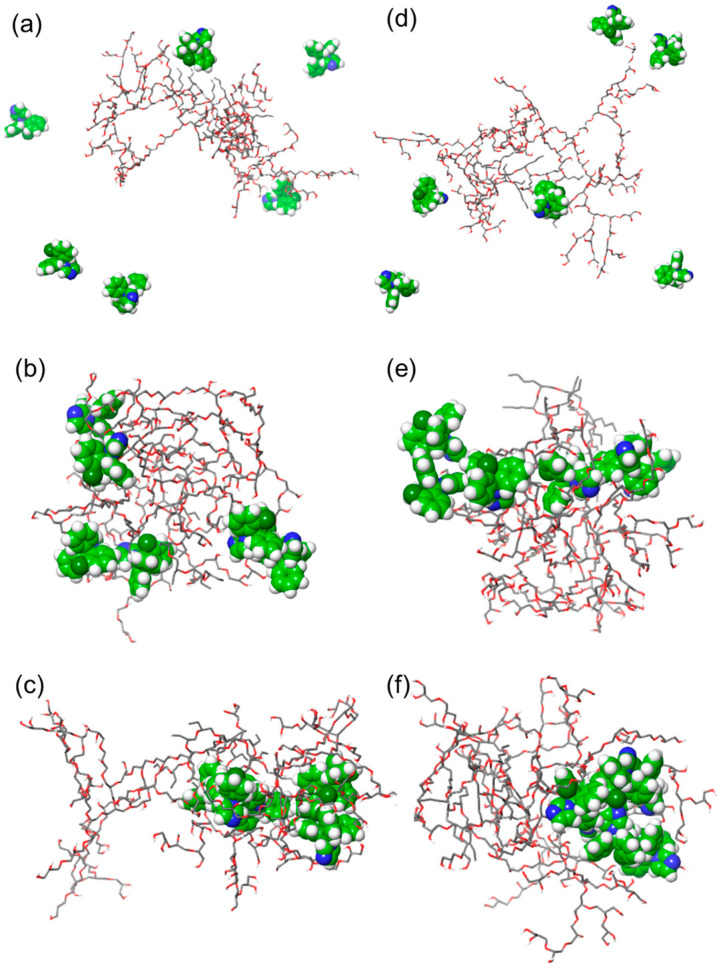
Final frames of MD simulation trajectories of a mixture of polymers and a hydrophobic drug. The R14 with six clotrimazole molecules was simulated in the following conditions: (**a**) dissolution in methanol (NPT simulation for 20 ns), (**b**) dried by removing methanol in four steps (NVT simulation for 10 ns), and (**c**) redissolved in water (NPT simulation for 100 ns). The R17 with six clotrimazole molecules was simulated with the following conditions: (**d**) dissolution in methanol (NPT simulation for 20 ns), (**e**) dried by removing methanol in four steps (NVT simulation for 10 ns), and (**f**) redissolved in water (NPT simulation for 100 ns).

Encapsulation of clotrimazole was carried out by two different polymers, R14 and R17, using the previously described MD simulation protocol. Clotrimazole is a hydrophobic drug (Xlog = 5) that is soluble in organic solvents and sparingly soluble in water. The simulations comprising one polymer molecule and six drug molecules were solvated in methanol and subjected to 20 ns MD simulations using an NPT ensemble. The polymers behaved in a similar manner as previously observed for a pure polymer by swelling as methanol molecules were breaking the intramolecular interactions and allowing the unfolding of the branches. The drug molecules did not form notable interactions with either polymer (Figure 7a,d). Extending simulations to 50 ns did not notably change the polymers’ conformation or form new drug–polymer interactions. The drying process protocol was then simplified by removing methanol in four stages instead without adverse effects on the potential intermolecular interaction formation. The drying process resulted in a different distribution of drug molecules in relation to two different polymers. All six molecules were found on the surface of R14 forming three smaller clusters (Figure 7b). On the contrary, all six drug molecules formed a single cluster with some of those encapsulated into the R17 interior (Figure 7e). This is most likely due to the presence of monomers with a larger hydrophobic moiety in R17.

It is noteworthy that the MD simulations of the redissolution of these complexes in water indicated different patterns of drug distribution compared to the redissolution of tinidazole–R17 complexes. None of clotrimazole molecules broke away from the hyperbranched polymers, or the duration of 50 ns simulation of systems fully solvated with water. On the contrary, this was observed in the case of simulations extended to 100 ns. The arrangement of six drug molecules with respect to the polymer changed by forming single clusters that were encapsulated by either of the polymers (Figure 7c,d). Hydrophobic molecules favourably interacted with the hydrophobic core, rather than breaking away into the bulk water. This suggests that R14 and R17 could be drug carriers that have the potential for targeted delivery.

### 2.2. Experimental Validation

#### 2.2.1. R14 and R17 Synthesis and Characterization

The potential of the two copolymers for targeted delivery systems was further evaluated. The syntheses were carried out according to the scheme presented in Figure S6. The polymers were purified by precipitation and dialysis, dried, and fully characterized using NMR spectroscopy, gel permeation chromatography, and MALDI-TOF spectrometry. The applied synthetic strategy assured the formation of macromolecules with distinctive hydrophobic cores based on poly(n-alkyl epoxide) and hydrophilic shells based on hyperbranched polyglycidol domains (Table S1).

The analytical data for R14, R17, and all intermediates are shown in Figures S7–S14. The NMR spectra indicated high purity of synthesized polymers free of unreacted monomer, suggesting that the purified material was suitable for further drug encapsulation studies.

#### 2.2.2. Tinidazole and Clotrimazole Encapsulation Studies

We experimentally evaluated the potential of the star-hyperbranched amphiphilic constructs as carriers of hydrophobic drugs in the aqueous medium.

The encapsulation process of both tinidazole and clotrimazole was performed according to the solvent evaporation method using methanol. After the complete removal of methanol, the drug–polymer mixture was suspended in deionized water and filtrated to remove the fraction of the drug that was not encapsulated. Based on the encapsulation experiments performed, there was no apparent effect of the size of hydrophobic residue in the poly(n-alkyl epoxide) used on polymer encapsulation efficiency. The ^1^H-^1^H ROESY NMR spectra recorded in methanol for clotrimazole with HbPGL-based homopolymer and copolymers (Figures S21–S26) revealed cross-peaks with polyether backbone (around 3.5 ppm) with no apparent cross-peaks of drug hydrogen atoms and hydrogen atoms of alkyl groups in the range between 0.5 and 2.00 ppm (Figures S21–S26). These suggest that drug molecules interacted with the hydrophilic outer area of the copolyether constructs in methanol, which was consistent with the MD studies. The same behavior was observed for tinidazole, i.e., no cross-peaks were observed between the hydrophobic core of the copolymers and tinidazole, and only weak interactions between the polyether backbone of the HbPGL domain and tinidazole were visible in methanol (Figures S17–S20).

The experimental study supported by molecular dynamics investigations revealed that in the encapsulation process of both drugs, the incubation time of the drug with the polymer in methanol does not contribute significantly. In fact, the process of drug encapsulation occurs mostly due to the evaporation of methanol molecules. Upon the removal of methanol, the drug molecules begin to interact with the polymer matrix, and these interactions are enhanced when the drug–polymer mixture is suspended in water.

The encapsulation efficiency for both copolymers is significant and ranges from 88% to 93% in the case of encapsulating tinidazole (Table 1) as well as from 83% to 90% when mixtures of copolymers and clotrimazole were tested (Table 2).

It is notable that copolymers could encapsulate a higher number of tinidazole molecules (up to 22 molecules per R14 molecule) than clotrimazole (up to 9 molecules per molecule of R14). One of the possible reasons could be the size difference of two drug molecules, (247.27 g/mol vs. 344.84 g/mol). However, it is more likely that the differences in the mode of binding could affect the encapsulation efficiency. The tinidazole molecules were predicted to form small clusters and bind on the hydrophilic surfaces of the copolymers (Figure 5), while the clotrimazole molecule clusters tended to interact with the smaller surface of hydrophobic monomers in the core (Figure 6).

In silico studies have a wide range of applications in developing polymer-based drug delivery systems and formulations, including predicting the stability and conformations of the drug–polymer complex, determining the solubilization capacity of the polymer complex for poorly soluble drugs, modeling the release kinetics of the drug from the polymer complex, and mapping the free energy and diffusion pathways for drug loading and release.

Several studies have explored encapsulating tinidazole in various systems to improve its bioavailability and therapeutic efficacy. Encapsulation protects tinidazole from degradation in the gastrointestinal tract and enhances its uptake and circulation time [21,22,23]. Similarly, encapsulation of the clotrimazole was studied extensively [24,25,26,27], with some requiring complex preparations of formulations. Despite their importance as therapeutic agents and the number of different systems for their encapsulation, the range and scope of relevant in silico investigations of formulations are limited.

The solubilization of tinidazole in various mono-solvents was studied using molecular dynamics simulations to evaluate solute–solvent interactions at the atomistic level [28,29]. In more complex systems, the interactions of tinidazole with hydroxyapatite nanoparticles showed that the binding energy of tinidazole on HAp is much lower than that observed for doxorubicin due to the different chemical structures of the two drugs [30,31].

An example of the use of molecular dynamics of clotrimazole for predictive purposes is demonstrated by identifying compatible lipid excipients for three drugs. For each pair of three drugs and five lipid excipients, a 10 ns NVT simulation was conducted using the OPLS-AA force field to evaluate the composition of formed nanoparticles. The simulations albeit short were suitable for screening and formulation design and led to results that were in agreement with experimental results, which confirmed that in silico selection of lipid excipients was appropriate for further applications [32].

It has to be considered that simulation systems can be trapped in metastable states and that the short simulation may not adequately explore the polymer and small molecule interactions. Additional longer simulations (500 ns) were conducted for three different systems, and the final frames were compared to the final frames of previously acquired 50 ns MD simulations: polymers in explicit water (Figure S1), polymer clotrimazole mixtures in water (Figures S3 and S4), and final stages of drying mixture (methanol removal) (Figure S5). The observed polymer conformations and clotrimazole distributions with respect to the polymers are comparable. The flexibility of the polymers led to changes in their shapes as reflected through the variation of RG (Figure 2a,b and Figure S2); similar trends are observed for simulations at 50 ns and 500 ns confirming that the dynamic nature of polymers conformation and interactions with drug molecules can be evaluated using shorter simulations.

The choice of the force field and water model can also affect the outcomes of the simulation; therefore, we conducted additional simulations of polymer clotrimazole mixtures solvated in explicit water represented by the TIP3P model. The final frames of these simulations (Figures S3c,f and S4c,f) are not significantly different compared to conformations observed in simulations using SPC water model, suggesting that the choice of water model did not have a significant effect on the observed trends for the interactions between polymer and drug molecules in this study.

This study shows the potential of the MD simulations for encapsulation studies, and it is validated for these particular systems. It has to be stressed that similar validations (including the duration of simulations, choice of force field, and water model) should be conducted if different classes of polymers are used for the encapsulation.

## 3. Materials and Methods

### 3.1. Materials

1,1,1-tris(hydroxymethyl)propane (TMP), 1,2-epoxybutane, 1,2-epoxyhexane, and glycidol were purchased from Sigma-Aldrich (St. Louis, MO, USA). Sodium hydride was obtained by washing the 60 wt% dispersion in mineral oil (Sigma-Aldrich) with dioxane. Tinidazole was purchased from Ambeed (Arlington Heights, IL, USA) and used as received.

### 3.2. Synthesis of Poly(1,2-epoxybutane)-co-HbPGL (R14)

An amount of 0.300 g (2.24 mmol) of TMP was dried by azeotropic distillation with benzene. TMP was partially deprotonated (20%) with sodium hydride in dry THF. The reaction was carried out at 40 °C under reduced pressure overnight. THF was removed, and the formed alkoxide was dissolved in dry DMSO. 1,2-epoxybutane (3.23 g; 44.8 mmol) was distilled under vacuum into alkoxide. The polymerization was then performed at 50 °C under reduced pressure for 5 days. The polymer was extracted with hexane from the reaction mixture yielding the purified product (R10), which was analyzed with ^1^H NMR spectroscopy, MALDI-TOF, and GPC. DP_n_ = 3 × 5 (MALDI-TOF); M_n_ = 1700 and M_w_/M_n_ = 1.07 (GPC).

A sample of poly(1,2-epoxybutane), R10 (0.5 g; 3.88 × 10^−4^ mol), was dried by azeotropic distillation with benzene. An amount of 20 mol% of terminal OH groups was converted into alcoholates in the reaction with NaH in dry THF. The mixture was kept at 40 °C overnight in an argon atmosphere. Then, THF was removed, a polymer with alkoxide ends was dissolved in DMSO, and glycidol was dropped at the rate of 0.5 mL/h. The reaction was carried out under argon for another 24 h. The reaction was subsequently terminated by exposure to air. The reaction mixture dissolved in MeOH and precipitated in acetone was used to isolate the crude product. The crude product was re-dissolved in DMSO and dialyzed against DMSO for 48 h using the dialysis tube (MWCO = 3.5 kDa). The purified product was analyzed via ^1^H NMR, ^13^C NMR INVGATE spectroscopy, and GPC (M_n_ = 15,700, M_w_/M_n_ = 1.63). The characteristics of a product including a degree of branching (DB), a degree of polymerization (DP_n_) along with the molecular weight number based on ^13^C INVGATE NMR spectrum are given in Table S1.

### 3.3. Synthesis of Poly(1,2-epoxyhexane)-co-HbPGL (R17)

An amount of 0.500 g (3.73 mmol) of TMP was dried by azeotropic distillation with benzene. A mixture of 60 mg (2.5 mmol) of NaH in TMP was suspended in dry THF. The reaction was carried out under reduced pressure at 40 °C overnight. The formed alkoxide obtained by THF removal was dissolved in dry DMSO. The distillation of 1,2-epoxyhexane under vacuum resulted in alkoxide. The polymerization was performed at 50 °C under reduced pressure for 5 days. Then, the polymer was extracted from the reaction mixture using hexane. The collected hexane layers were evaporated, and the product (R13) was analyzed with ^1^H NMR spectroscopy, MALDI-TOF, and GPC. DP_n_ = 3 × 5 (MALDI-TOF); M_n_ = 1940 and M_w_/M_n_ = 1.13 (GPC).

A sample of poly(1,2-epoxyhexane), R13 (0.674 g; 3.88 × 10^−4^ mol), was dried by washing with 15 mL benzene, which was subsequently removed under vacuum. An amount of 7.2 mg of NaH was added, and THF was distilled. The mixture was kept at 40 °C overnight under an argon atmosphere. Then, THF was removed, a polymer with alkoxide ends was dissolved in DMSO, and glycidol was dropped at a rate equal to 0.5 mL/h. The reaction was carried out under argon for 24 h. The reaction was subsequently terminated by exposure to air. The crude product was extracted by dissolution of the reaction mixture in MeOH followed by precipitation by acetone. The isolated crude product was re-dissolved in DMSO and dialyzed for 48 h using the dialysis tube (MWCO = 3.5 kDa). The purified product was analyzed with ^1^H NMR, ^13^C NMR INVGATE spectroscopy, and GPC (M_n_ = 8500, M_w_/M_n_ = 1.98). The characteristics of a product including a degree of branching (DB), a degree of polymerization (DP_n_) along with the molecular weight number based on ^13^C INVGATE NMR spectrum are given in Table S1.

### 3.4. Gel Permeation Chromatography, GPC

The average molecular weights (M_n_) of R10 and R13 copolymers were determined using a GPC system comprising (Agilent G1379A Degasser coupled to Agilent Pump 1100 Series), two PLGel 5 μMIXED-C columns, and two different laser photometers as detectors, namely Wyatt Optilab Rex differential refractometer and Dawn Eos (Wyatt Technology Corporation, Santa Barbara, CA, USA). The elution was carried out using dichloromethane at a flow rate of 0.8 mL min^−1^ at room temperature. The average molecular weights (M_n_) of R14 and R17 copolymers were evaluated using an aqueous solution of NaN_3_ (0.1 wt%) as eluent at a flow rate of 1.0 mL/min and chromatograph comprising Knauer K-501 HPLC pump and a degasser (4-Channel Degasser; K-5004, Knauer, Biberach, Germany). Three different TSK-GEL columns, G5000 PW_XL_ + 3000 PW_XL_ + 2500 PW_XL_ (7.8 × 300 mm^2^; Tosho; 26 °C), were used coupled to an LDC RI detector, and a Viscotek Dual Detector, Malvern Panalytical, Malvern, United Kingom, (laser light scattering at λ = 670 nm (RALS and LALS) and a differential viscometer). RALS measurements and viscometry principles were utilized to calculate the average molecular weights of all copolymers.

### 3.5. Matrix-Assisted Laser Desorption/Ionization Time-of-Flight Mass Spectrometry, MALDI-TOF

The mass spectra of both R14 and R17 copolymers were obtained using MALDI-TOF/TOF Axima Performance Mass Spectrometer, Shimadzu Biotech (Shimadzu, Kyoto, Japan). The samples were prepared in dichloromethane at concentrations of 5 mg/L. A volume of 20 μL of copolymer solutions was mixed with 10 μL of ditranol and 1 μL of potassium salt solutions in THF previously prepared at 8 mg/L concentrations. The mass spectra of dried samples were recorded in the linear positive mode using an accelerating voltage of 5.6 mV. Each spectrum had an average of 200 scans.

### 3.6. Evalulation of Drug Loadings in Copolymers

Stock solutions of clotrimazole and tinidazole in methanol were prepared at 5 mg/mL concentrations. Selected weights of copolymers were dissolved in 2 mL of methanol and mixed with stock solutions of drug molecules to make four different copolymer–drug mixtures (Table 3), which were stirred for 30 min. The samples were subsequently dried at 35 °C overnight. The dry polymer–drug samples were dissolved in 3 mL of deionized water, the suspension was filtered using a 0.45 µm PTFE syringe filter, and the clear solution was lyophilized overnight. The drug loadings in the copolymers for each sample were calculated using the integration of selected peaks in ^1^H NMR spectra of drugs dissolved in DMSO-d_6_ compared to the DMF peak added as the internal standard.

### 3.7. Generation of 3D Structures

The structures of drug molecules were obtained by downloading from the PubChem website using VegaZZ (version 3.2.3.28) as a graphic user interface. The hydrogen atoms of both molecules were added to reflect protonation states of ionizable groups at pH 7.4 and minimized using AMMP molecular mechanics software (version 2.4.0) and the genetic algorithm minimization approach implemented in VegaZZ. The final 3D structures were saved in mol2 file format.

The 3D structures of start-hyperbranched polymers were generated using an in-house Python script for the automatic generation of fully atomistic hyperbranched polymers from monomers that take into account the polydispersity and degree of branching of the polymers obtained experimentally. Our Python script generated a population of possible polymers using a set of constraints. One of the constraints was the total number of monomers, which was set according to the target molecular weight of the polymer. The quantity of each monomer, also set as a constraint, was created based on the distribution determined experimentally. Each monomer type and the core were then converted into SMILES using RDKit (version 2023.03.02), and a substitution point was added to the core and non-terminal monomers, where further monomers could be inserted to grow the polymer. The growth of the polymer by expansion from the core polymer was carried out by randomly picking the different monomers from the set of monomers available. The selection of terminal monomers was limited when the ramification ratio was inferior to 0.5 or when the ratio of non-terminal monomers was inferior to 0.5. This prevented undesired early capping of the branches, which ultimately made it impossible to generate a polymer with the target molecular weight. Only solutions of polymers that use all monomers were made available at the start (therefore obeying the desired monomer distribution), and a desired (i.e., experimentally derived) polymerization degree was generated, in the form of SMILES. We then converted the SMILES to 2D structures using RDKit and saved them as PDB files. Using OpenBabel, hydrogen atoms were then added, 3D coordinates were generated to produce structures that are suited for molecular dynamics simulations, and the final conformation was saved in a PDB file format.

### 3.8. Molecular Dynamics Simulations

Molecular dynamics simulations in this study were carried out using Desmond molecular dynamics simulation software (version 2022.4) andOPLS2005 all atoms force field [33], with the Maestro version as a graphical user interface (Schrödinger, New York, NY, USA). The system builder tool was utilized to fully solvate the final conformations of copolymers using a cubic periodic system and two different water models (SPC and TIP3P models). All systems for simulation were built with sides 15 Å larger than copolymers in all directions with 0.15 M NaCl concentration, including additional Na^+^ and Cl^−^ as counter ions if needed. The default simulated annealing was modified by adding an additional step of simulation of the system for 1000 ps at 300 K. The cut-off at 9.0 Å was set by default for short-range Coulombic interactions. The Berendsen thermostats, with 1.0 ps relaxation time, were used to regulate simulation temperature within the NVT ensemble. The final frames of generated trajectories were used as starting points for further 50 ns and 500 ns NPT simulations.

The tinidazole encapsulation by R17 was conducted by building molecular systems comprising one polymer and six drug molecules randomly positioned around the polymer. System builders were used to generate fully solvated systems by using three different solvent models water, methanol, and DMSO, and each of the systems was simulated for 20 ns. Finally, the final frame of the simulated system in methanol was subjected to a drying protocol, where the solvent molecules were removed in stages according to Table 4. For each stage of drying, the solvent was kept within the shell around all polymer and drug molecules. The molecular system obtained after simulation at stage 7 was used to generate a fully solvated system by adding water molecules and running an NPT simulation for 50 ns.

The clotrimazole encapsulation by R14 and R17 was conducted in a similar manner with fewer steps to mimic drying (Table 5).

## 4. Conclusions

The rapid growth of computational power and modeling algorithms has propelled the development of in silico methods, including molecular dynamics, for the design of pharmaceutical formulations. These methods can be a valuable complement to traditional formulation screening. The results of this study indicate that with currently available software, adaptations of protocols can be used to mimic not only compositions of the formulations but also some simple processes used in formulation development. Further research is essential to optimize in silico composition screening in a high-throughput manner.

## Figures and Tables

**Table 1:** Encapsulation data of tinidazole within star-hyperbranched constructs based on poly(n-alkyl epoxide)-*co*-HbPGL.

	Poly(1,2-epoxybutane)-*co*-HbPGL		Poly(1,2-epoxyhexane)-*co*-HbPGL
Tinidazole aimed (per copolymer molecule)	15	25	15
Tinidazole encapsulated	14	22	14
Encapsulation efficiency, %	93	88	93

**Table 2:** Encapsulation data of clotrimazole within star-hyperbranched constructs based on poly(n-alkyl epoxide)-*co*-HbPGL.

	Poly(1,2-epoxybutane)-*co*-HbPGL	Poly(1,2-epoxyhexane)-*co*-HbPGL
Clotrimazole aimed (per copolymer molecule)	10	6
Clotrimazole encapsulated	9	5
Encapsulation efficiency, %	90	83

**Table 3:** The composition of copolymer/drug mixture used for drug encapsulation.

	System 1	System 2	System 3	System 4
Copolymer	R14	R14	R17	R17
Weight of copolymer (mg)	42.5	30	46.5	30
Drug	clotrimazole	tinidazole	clotrimazole	tinidazole
Volume of drug solution (mL)	3	from 2.25 to 3.70	2	2.25

**Table 4:** General protocol for simulation of creating R17–tinidazole mixture, the gradual removing of methanol molecules from the molecular systems to mimic formulation drying and reconstitution of formed complexes.

Stage	Stage Name	Solvent	Solvent Shell (Å)	Ensemble	Simulation (ns)
1	Dissolving	MeOH		NPT	20
2	Drying stage 1	MeOH	25	NVT	10
3	Drying stage 2	MeOH	20	NVT	10
4	Drying stage 1	MeOH	15	NVT	10
5	Drying stage 2	MeOH	10	NVT	10
6	Drying stage 1	MeOH	5	NVT	10
7	Drying stage 2	MeOH	2.5	NVT	10
8	Redissolving	Water		NPT	50

**Table 5:** A simplified protocol for simulation of creating R14–clotrimazole and R17–clotrimazole mixture, the gradual removing methanol molecules from the to mimic formulation drying and reconstitution of formed complexes.

Stage	Stage Name	Solvent	Solvent Shell (Å)	Ensemble	Simulation (ns)
1	Dissolving	MeOH		NPT	20
2	Drying stage 1	MeOH	15	NVT	10
3	Drying stage 2	MeOH	6	NVT	10
4	Drying stage 1	MeOH	3	NVT	10
5	Drying stage 2	MeOH	2	NVT	10
6	Redissolving	Water		NPT	50

## Data Availability

The structural data of the modelled hyperbranched copolymers are available from authors on request.

## Supplementary Materials

The following supporting information can be downloaded at: https://www.mdpi.com/article/10.3390/molecules28217308/s1. In-house scripts for the generation of the R14 and R17 structures are available on request.
