# Supplementary material for: Evaluation of Encapsulation Potential of Selected Star-Hyperbranched Polyglycidol Architectures: Predictive Molecular Dynamics Simulations and Experimental Validation
Source: Molecules. 2023 Oct 28;28(21):7308. doi: 10.3390/molecules28217308 (PMC10650410; doi:10.3390/molecules28217308)
Supplement: Supplementary file 1 [file molecules-28-07308-s001.zip › molecules-2564844-supplementary.pdf]

Supplementary material

Evaluation of encapsulation potential of selected star-hyperbranched polyglycidol architectures: predictive molecular dynamics simulations and experimental validation

Mateusz Gosecki[1], Malgorzata Urbaniak[1], Nuno Marinho[2], Monika Gosecka[1*], and Mire Zloh[3,4,*]

1 Centre of Molecular and Macromolecular Studies, Polish Academy of Sciences, Sienkiewicza 112, 90-363 Lodz, Poland; mdybko@cbmm.lodz.pl
2 iBB—Institute for Bioengineering and Biosciences, and Associate Laboratory i4HB—Institute for Health and Bioeconomy at Instituto Superior Técnico, Universidade de Lisboa, Av. Rovisco Pais, 1049-001 Lisboa, Portugal
3 University College London, UCL School of Pharmacy, 29/39 Bruunswick Square, London WC1N 1AX, United Kigdom; m.zloh@ucl.ac.uk
4 University Business Academy, Faculty of Pharmacy, Trg Mladenaca 5, 21000 Novi Sad, Serbia
* Correspondence: MZ m.zloh@ucl.ac.uk; mdybko@cbmm.lodz.pl

Contents

In silico studies 2
- Link to movies of simulations 2
- Effects of duration of the MD simulations and choice of water model 3

Experimental validation section 8
- Synthesis conditions scheme 8
- Characterization of polymers and drug molecules 9
  - Characterization of R14 – spectral data 9
  - Characterization of R17 14
- ^{1}H NMR spectrum of tinidazole 17
  - Encapsulation tinidazole by R14 19
  - Encapsulation tinidazole by R17 21
  - Encapsulation clotrimazole by R14 23
  - Encapsulation clotrimazole by R17 25

Supplementary material

In silico studies

Link to movies of simulations

The movies generated from the trajectoris of the molecular dynamics simulations of the tinidazole encapsulation by gradual removing methanol can be found at the following shared folder:

https://drive.google.com/drive/folders/14CK6ATFT3vu0c5kxCwpWkuNp_bXnEE8r?usp=sharing

The movies should be viewed in the following order:

1-R17-6xtinidazole-MeOH_desmond_md_job_start.mpeg

2-R17-6xtinidazole-MeOH_desmond_md_job_25A.mpeg

3-R17-6xtinidazole-MeOH_desmond_md_job_20A.mpeg

4-R17-6xtinidazole-MeOH_desmond_md_job_15A.mpeg

5-R17-6xtinidazole-MeOH_desmond_md_job_10A.mpeg

6-R17-6xtinidazole-MeOH_desmond_md_job_5A.mpeg

7-R17-6xtinidazole-MeOH_desmond_md_job_2.5A.mpeg

8-R17-6xtinidazole_from_MeOH_redissolution_in_water_50.mpeg

Supplementary material

Effects of duration of the MD simulations and choice of water model

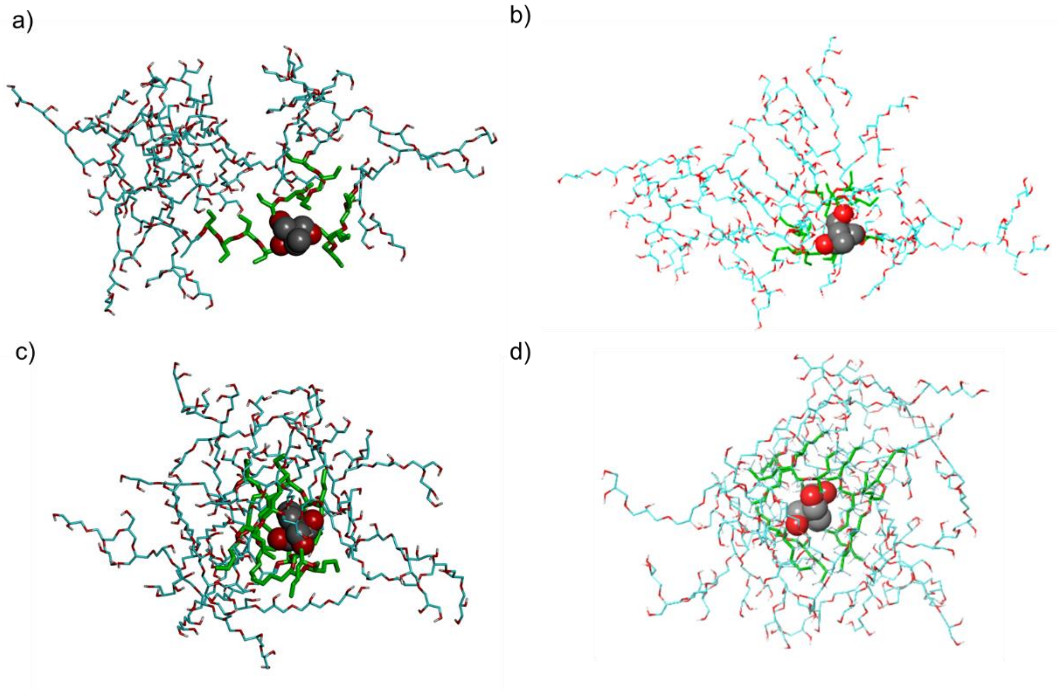

Figure S1. Final frames of the MD simulation trajectories of polymers based on HbPGL shown as a mixture of CPK and stick representation: R14 simulated for (a) 50 ns and (b) 500 ns; R17 systems simulated for (b) 50 ns and (d) 500 ns. The cores of the polymers are shown in CPK representation, hydrophobic polyether residues are shown as thick sticks coloured in green, and hydrophilic corona residues are shown as thin sticks coloured in cyan; only polar hydrogen atoms are shown for clarity.

Supplementary material

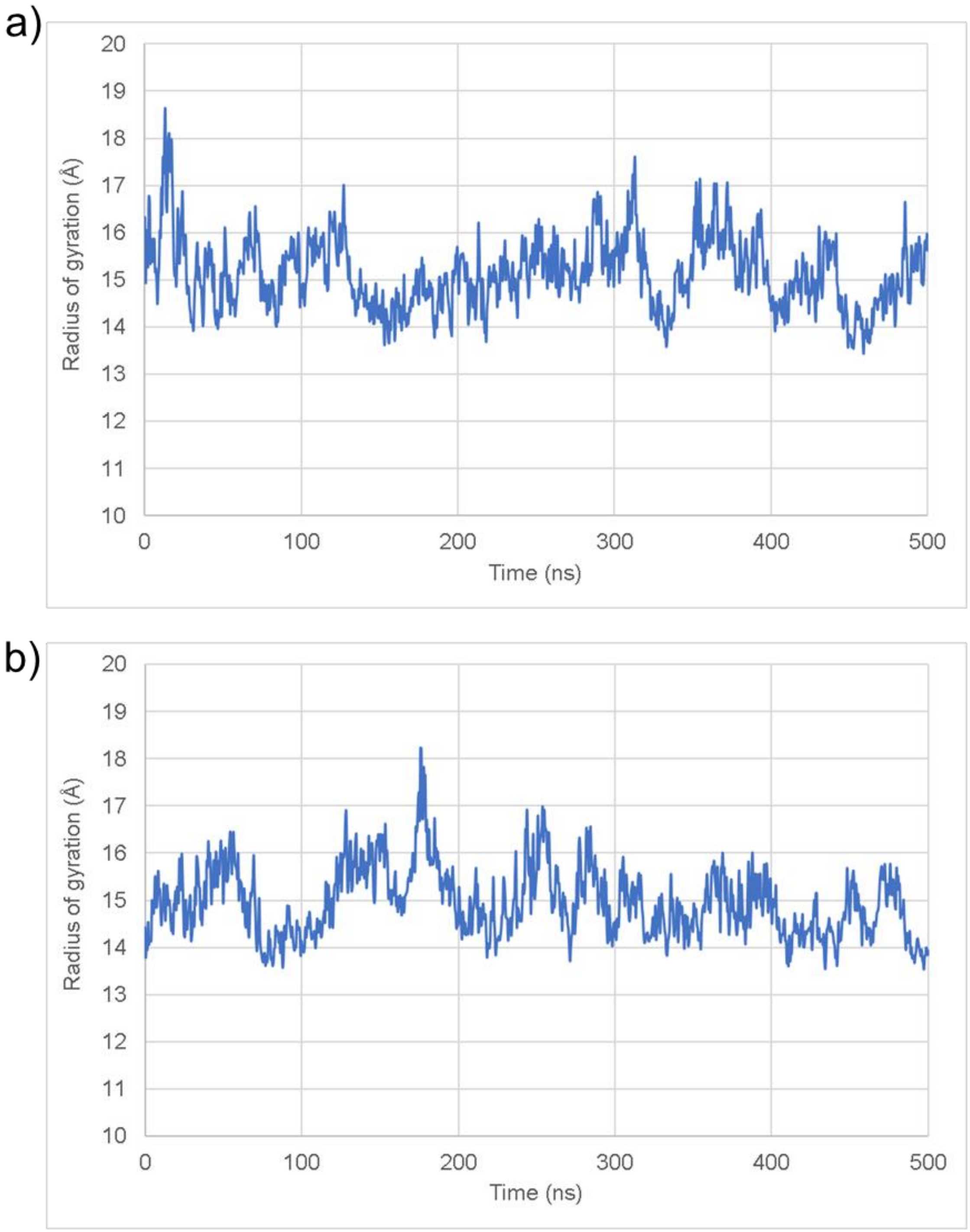

Figure S2. Radius of gyration as a macroscopic property of (a) R14 and (b) R17 calculated over 500 ns MD simulation at 300 K using Desmond software and OPLS-2005 force field. The average value of radius of gyration of R14 and R18 are 15.1 ± 0.8 Å and 14.9 ± 0.7 Å, respectively. These values are comparable to those observed in 50 ns MD simulations of five different species, The average value of radius of gyration of R14 and R18 are 15.5 ± 1 Å and 15.4 ± 1 Å, respectively.

Supplementary material

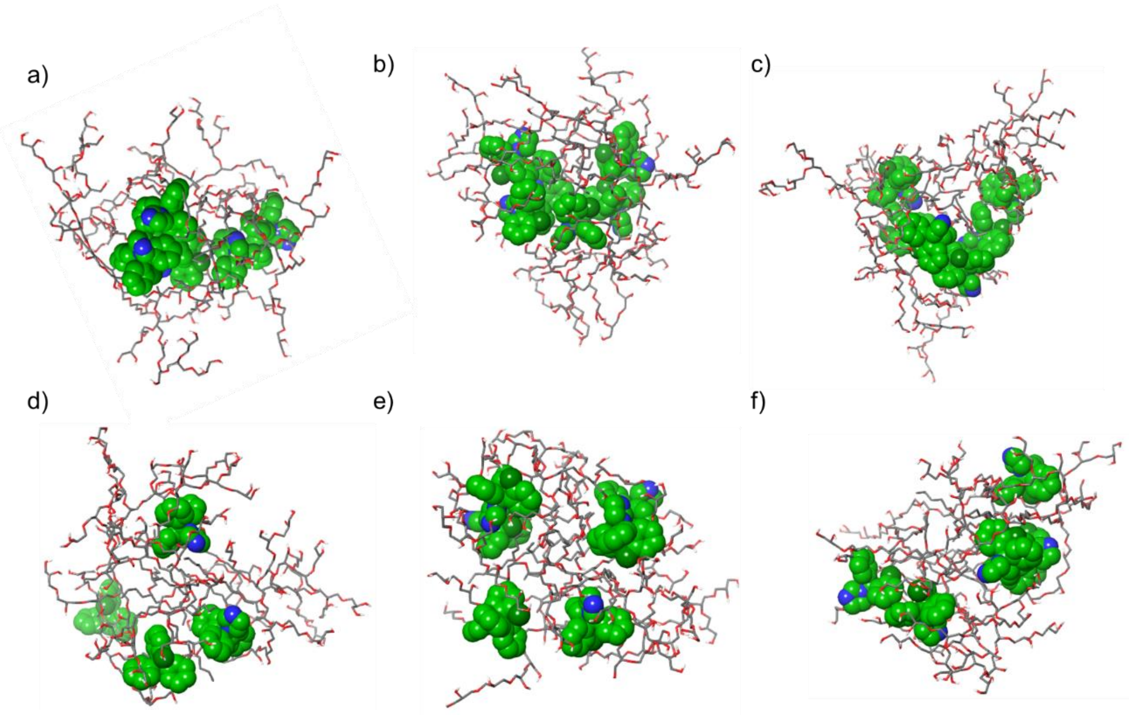

Figure S3. Final frames of MD simulation trajectories of a mixture of polymers and a hydrophobic drug comparing the effects of the duration of the simulation and water model. The ligand molecules were randomly positioned away from the polymer at the beginning of the simulation. All simulations were conducted using Demond software and OPLS force field.The R14 with 6 clotrimazole molecules were simulated in the following conditions: (a) SPC water model and 50 ns MD simulation, (b) SPC water model and 500 ns MD simulation and (c) TIP3P water model and 50 ns MD simulation. The R17 with 6 clotrimazole molecules were simulated in the following conditions: (d) SPC water model and 50 ns MD simulation, (e) SPC water model and 500 ns MD simulation and (f) TIP3P water model and 50 ns MD simulation.

Supplementary material

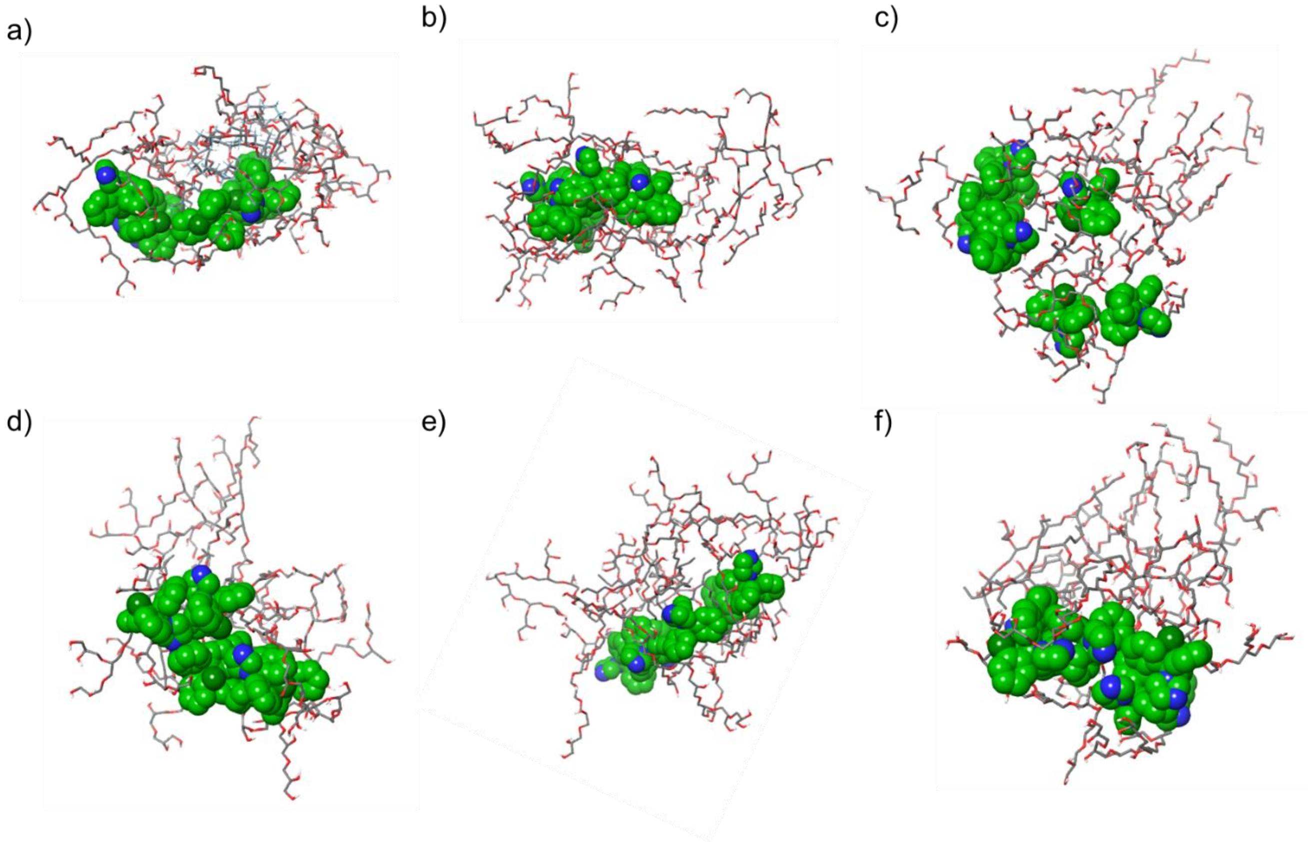

Figure S4. Final frames of MD simulation trajectories of a mixture of polymers and a hydrophobic drug comparing the effects of the duration of the simulation and water model. The final frames of the trajectories of the simulations of the systems after the methanol solvent removal (dried mixtures) were used for building molecular systems for simulation of redissolving process. All simulations were conducted using Demond software and OPLS force field.The R14 with 6 clotrimazole molecules were simulated in the following conditions: (a) SPC water model and 50 ns MD simulation, (b) SPC water model and 500 ns MD simulation and (c) TIP3P water model and 50 ns MD simulation. The R17 with 6 clotrimazole molecules were simulated in the following conditions: (d) SPC water model and 50 ns MD simulation, (e) SPC water model and 500 ns MD simulation and (f) TIP3P water model and 50 ns MD simulation.

Supplementary material

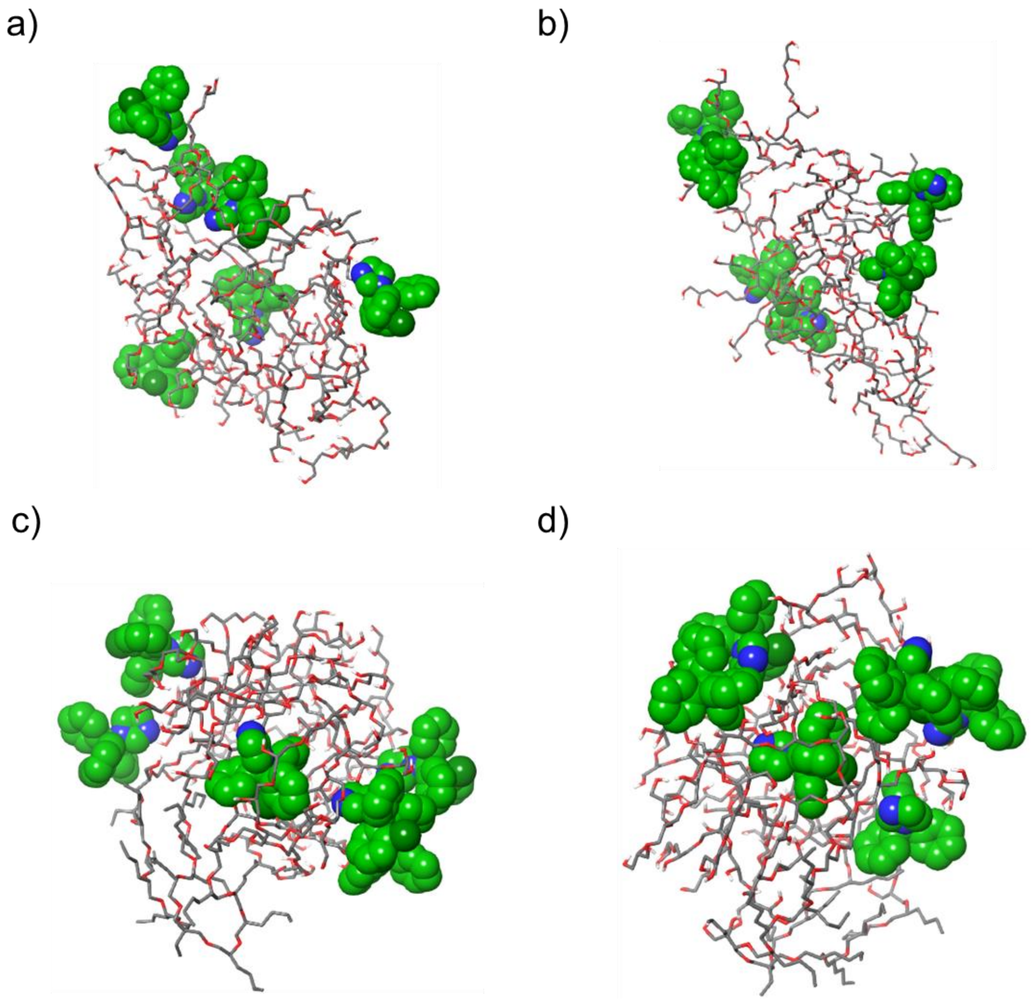

Figure S5. Final frames of MD simulation trajectories of a mixture of polymers and a hydrophobic drug at the end of the drying process (all methanol molecules beyond 2 Å were removed) comparing the effects of the duration of the simulation and water model. All simulations were conducted using Demond software, OPLS force field and methanol as explicit solvent.The R14 with 6 clotrimazole molecules were simulated in the following conditions: (a) 10 ns MD simulation (NVT system) and (b) 500 ns MD simulation (NVT system). The R17 with 6 clotrimazole molecules were simulated in the following conditions: (c) 10 ns MD simulation (NVT system), (d) 500 ns MD simulation (NVT system).

Supplementary material

Experimental validation section

Synthesis conditions scheme

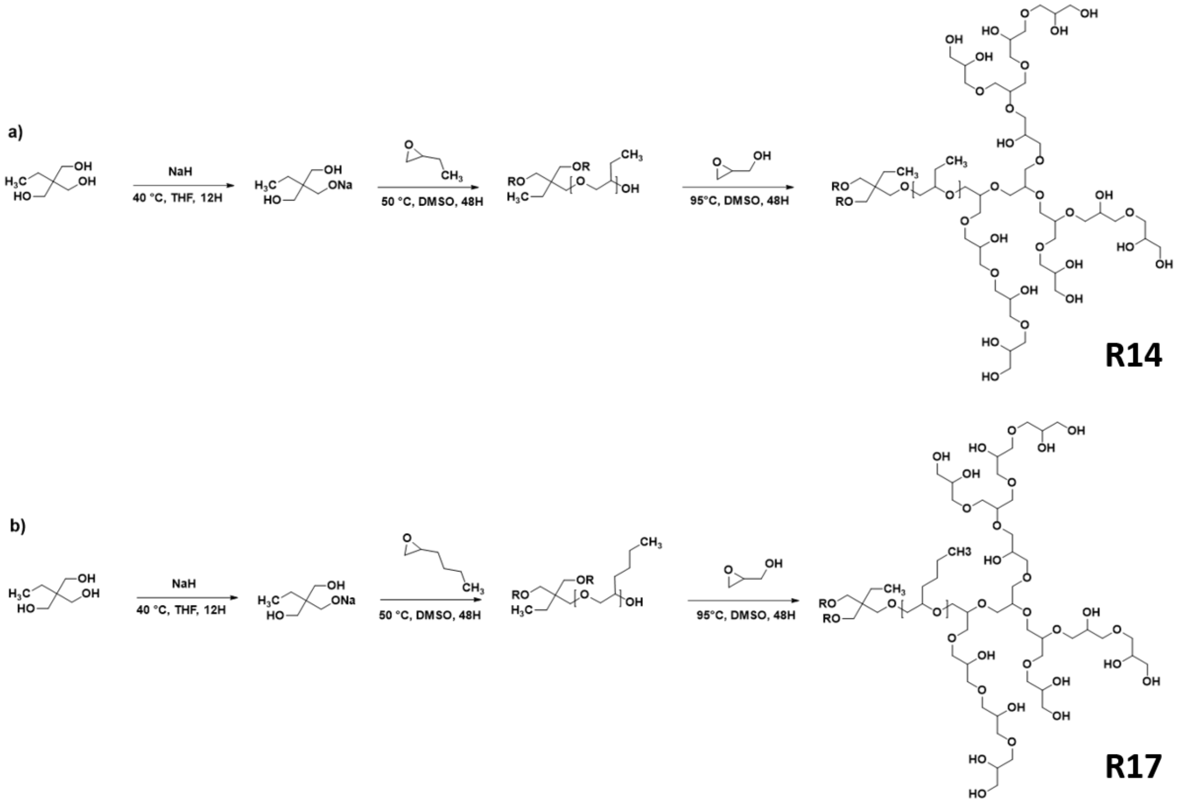

Figure S6: Schematic representation of the synthesis of (a) poly(1,2-epoxybutane)-*co*-HbPGL (**R14**) and (b) poly(1,2-epoxyhexane)-*co*-HbPGL (**R17)**.

Supplementary material

Characterization of polymers and drug molecules

Characterization of R14 – spectral data

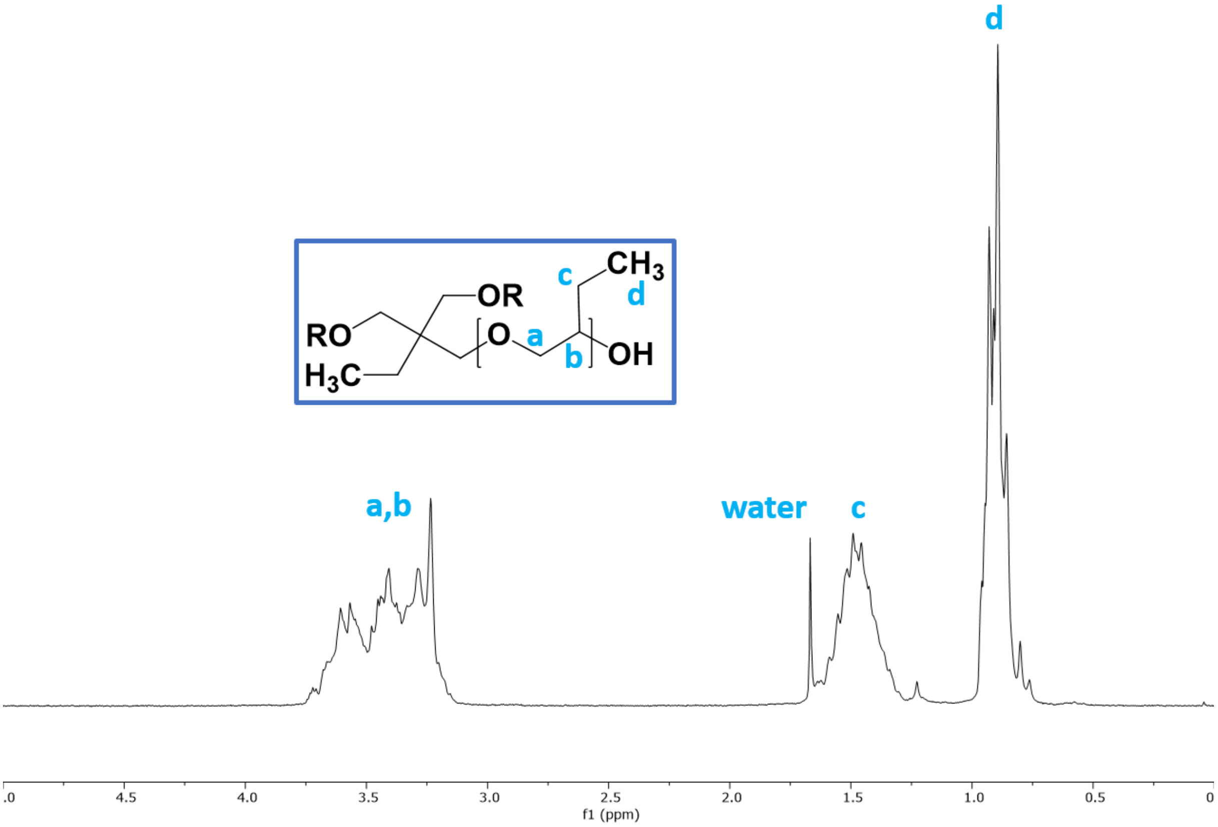

Figure S7. ^{1}H NMR spectrum of poly(1,2-epoxybutane), R10 recorded in $CDCl_3$.

Supplementary material

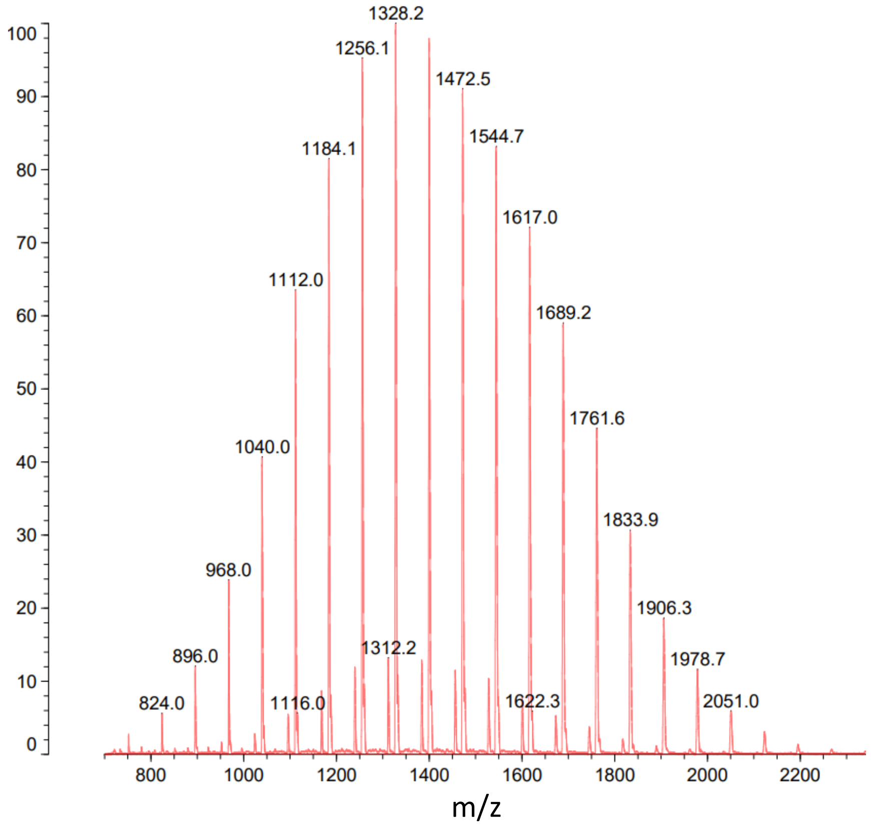

Figure S8. MALDI-TOF spectrum of poly(1,2-epoxybutane), R10.

Supplementary material

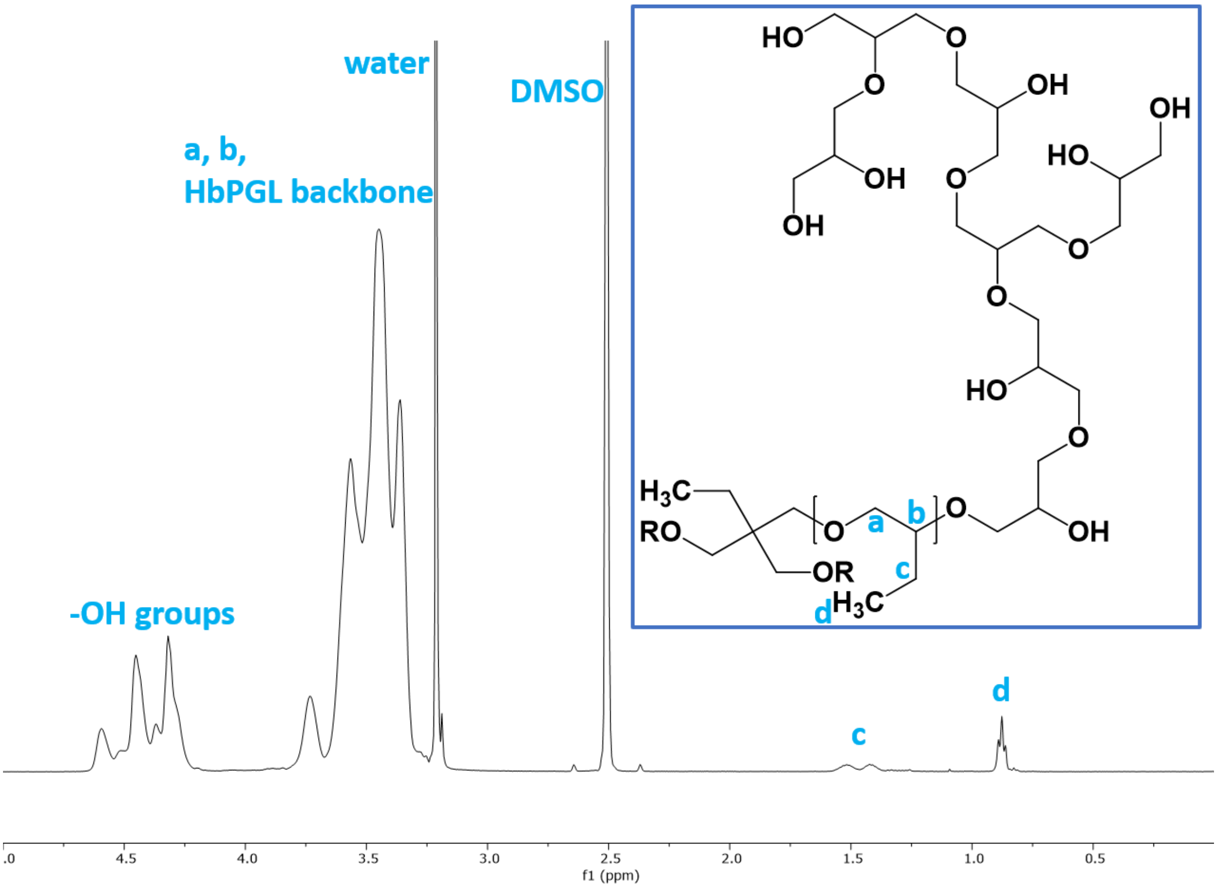

Figure S9. ^{1}H NMR spectrum of poly(1,2-epoxybutane)-*co*-HbPGL, R14 recorded in DMSO-*d6*.

Supplementary material

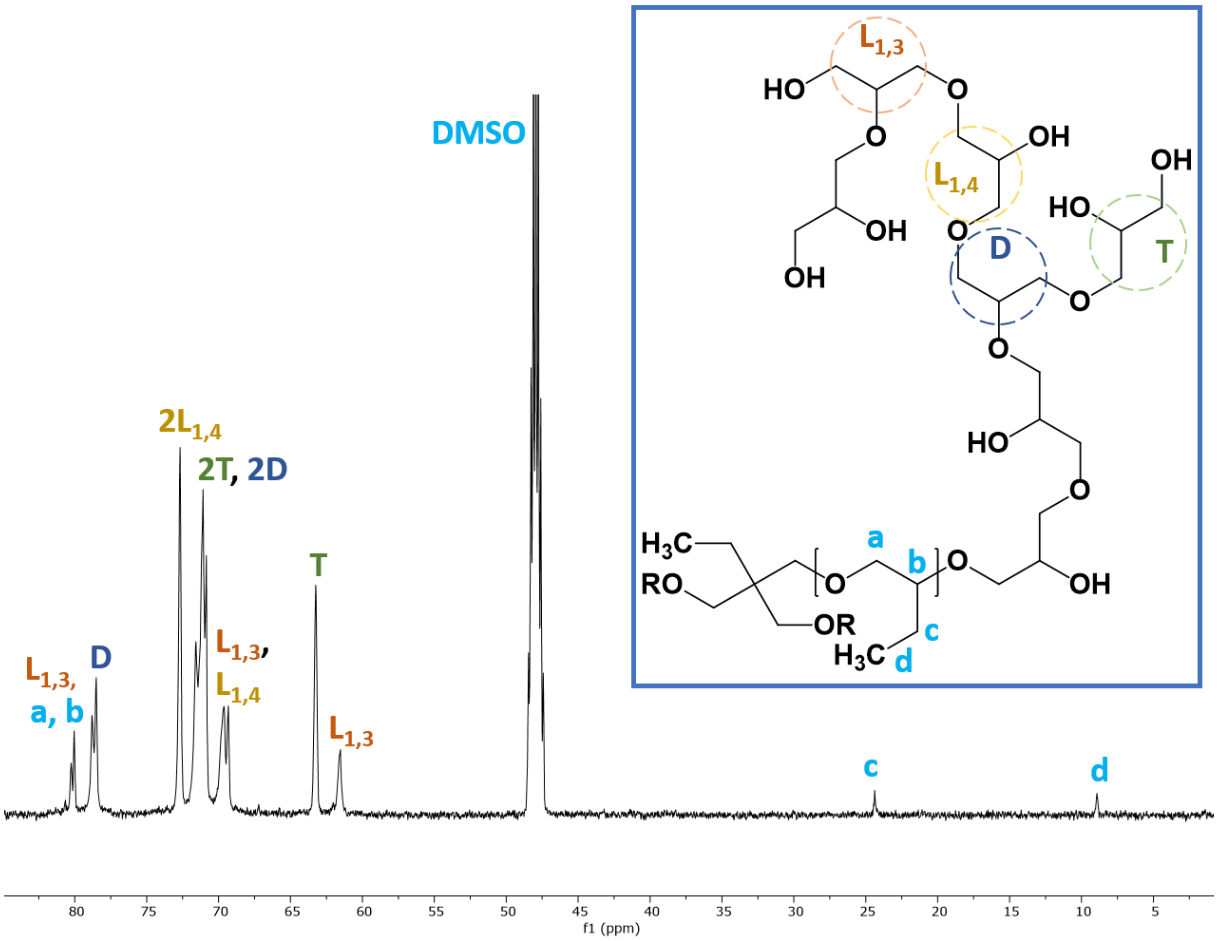

Figure S10. ^{13}C INVGATE NMR spectrum of poly(1,2-epoxybutane)-*co*-HbPGL, R14 recorded in DMSO-*d6*.

Supplementary material

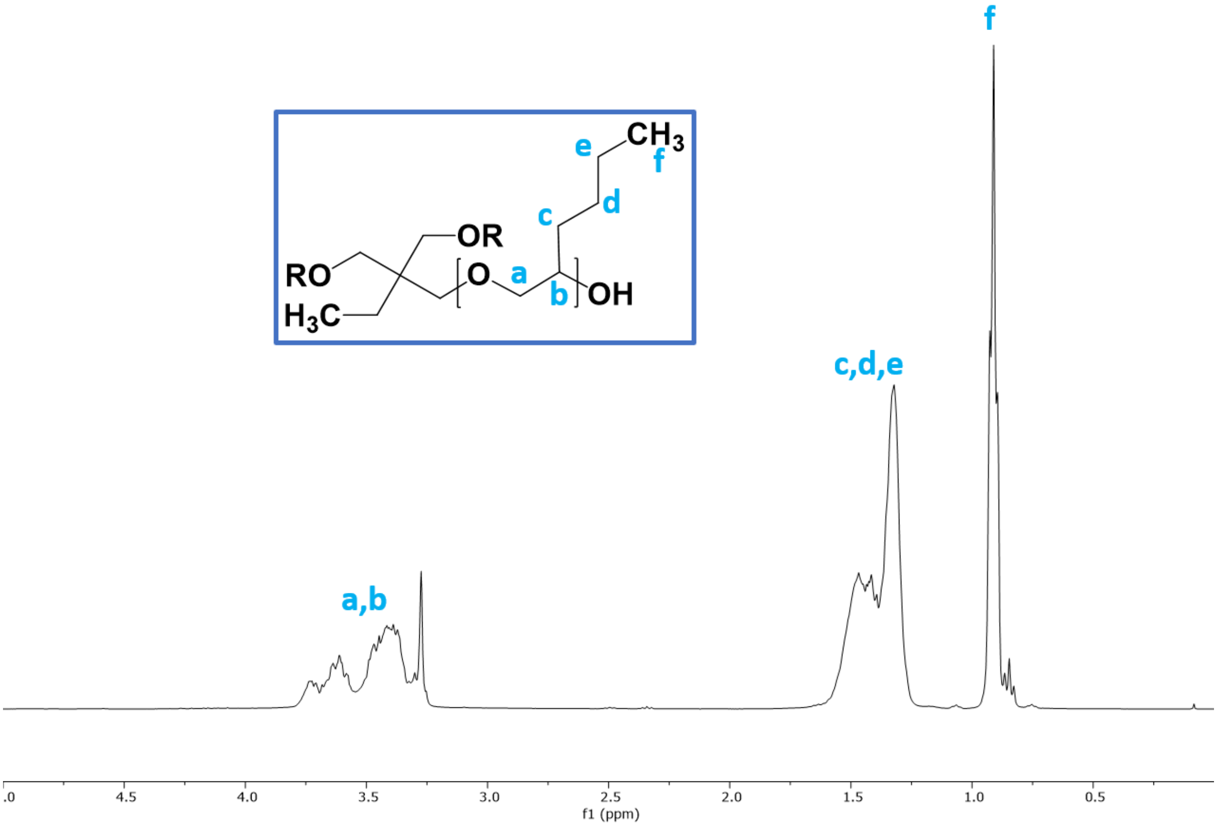

Figure S11. ^{1}H NMR spectrum of poly(1,2-epoxyhexane), R10 recorded in $CDCl_3$.

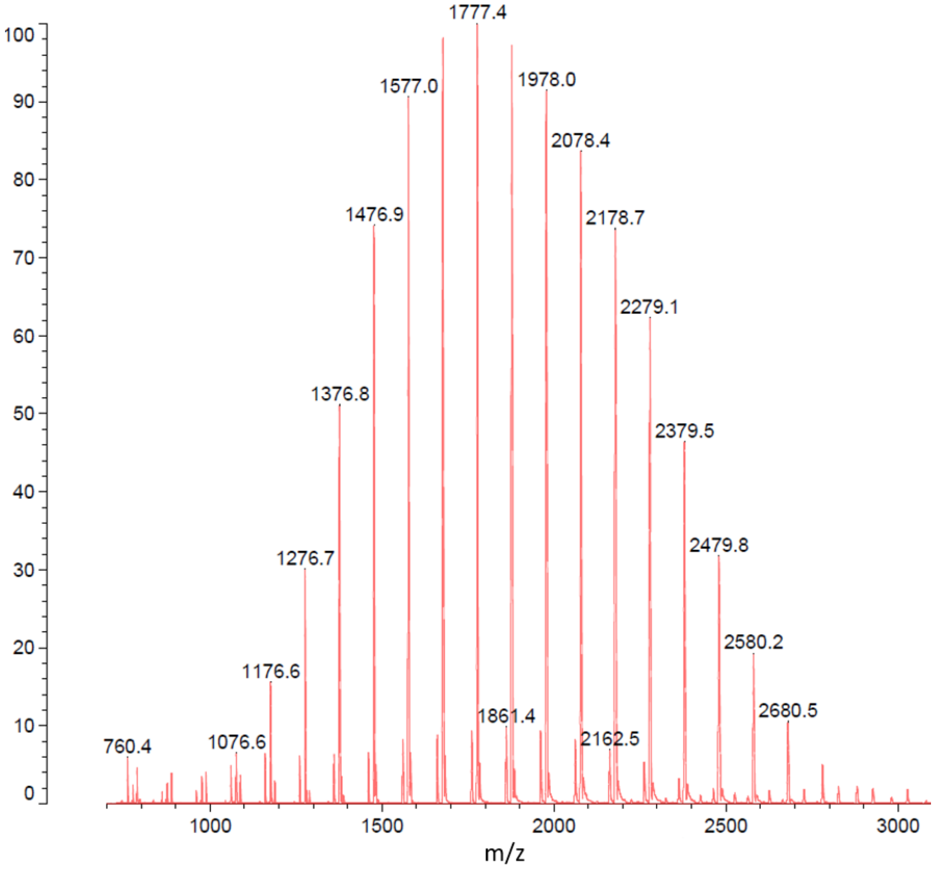

Figure S12. MALDI-TOF spectrum of poly(1,2-epoxyhexane), R13.

Supplementary material

Characterization of R17

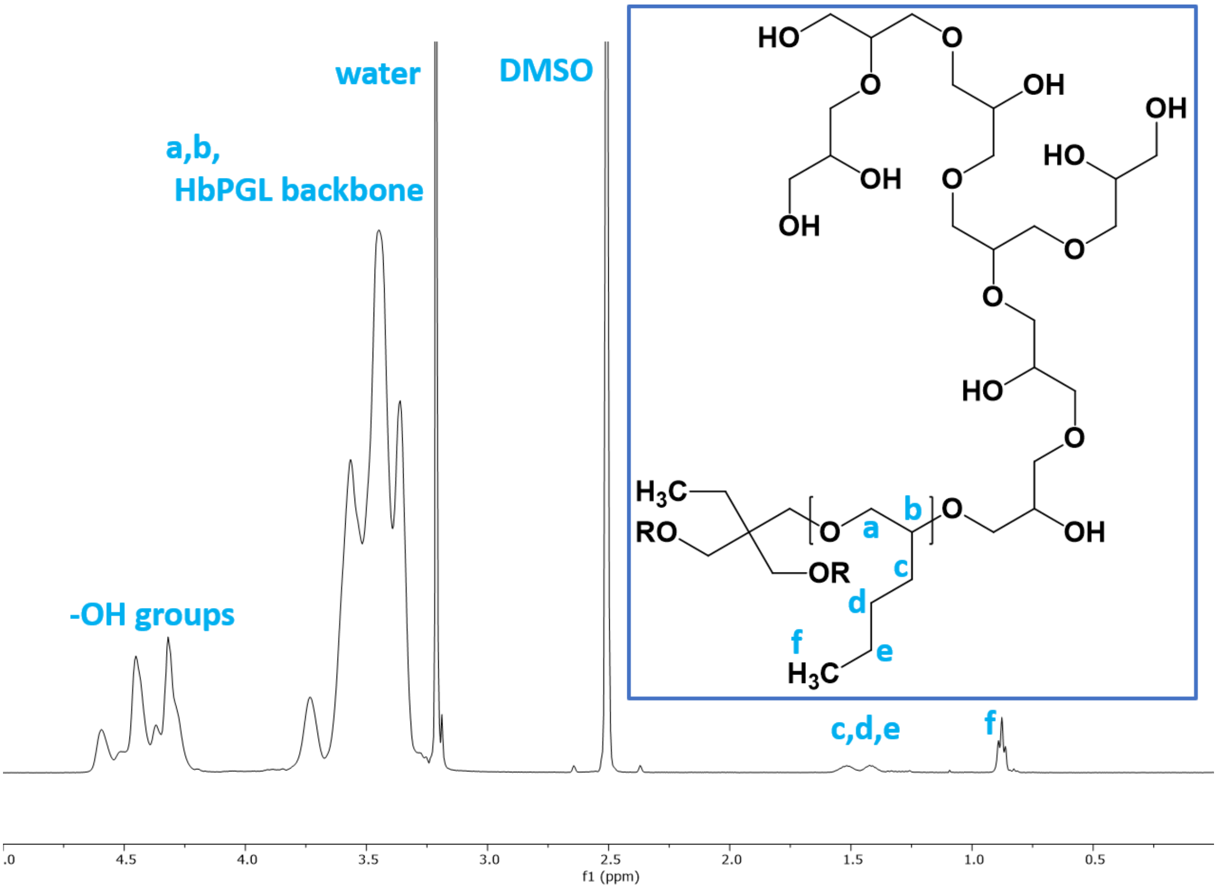

Figure S13. ^{1}H NMR spectrum of poly(1,2-epoxyhexane)-*co*-HbPGL, R17 recorded in DMSO-*d6*.

Supplementary material

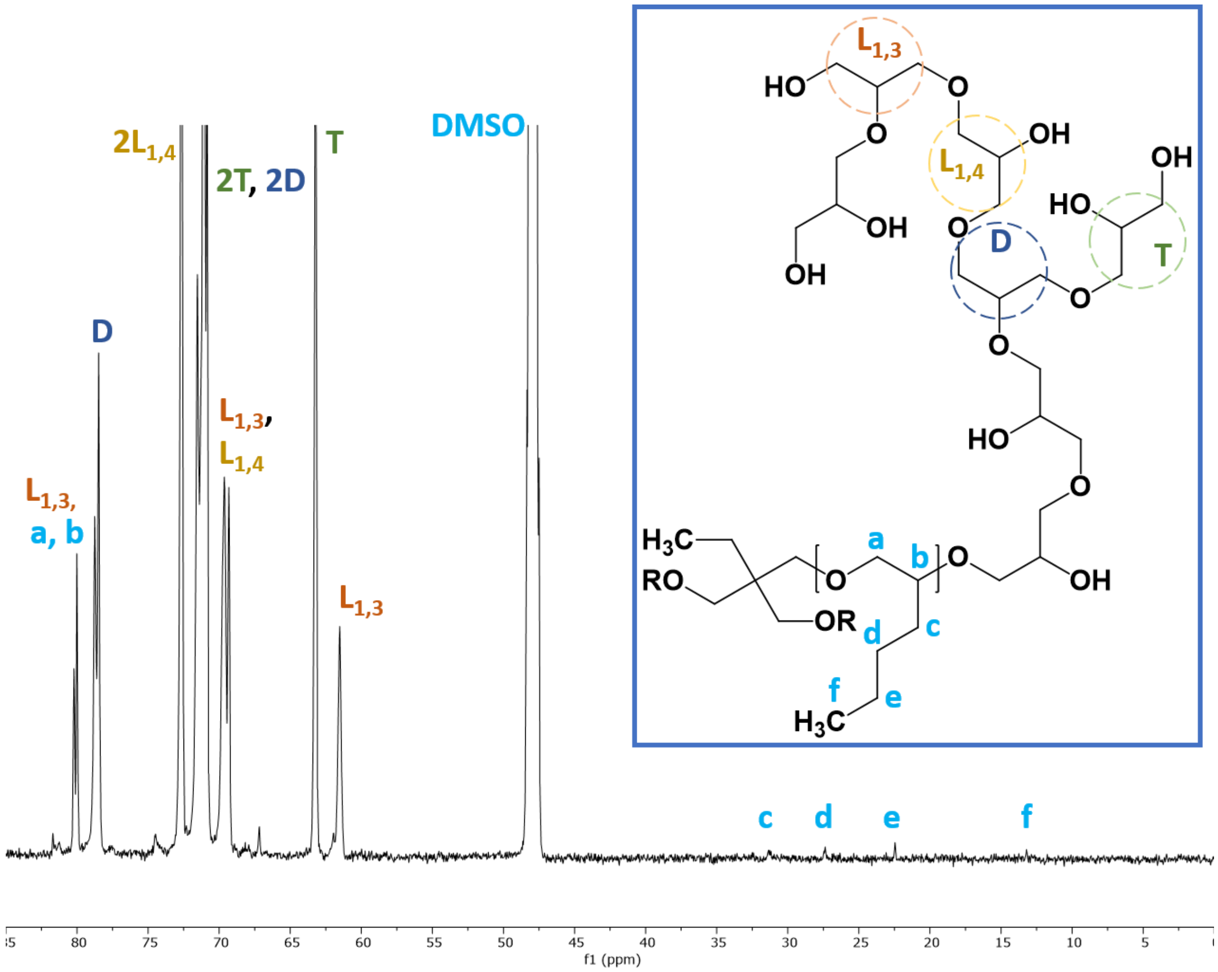

Figure S14. ^{13}C INVGATE NMR spectrum of poly(1,2-epoxyhexane)-*co*-HbPGL, R17 recorded in DMSO-d6.

Supplementary material

Table S1. Interpretation of ^{13}C NMR INVGATE spectra of amphiphilic copolymers.

Copolymer	Molar fraction of HbPGL's repeating units				DB	DP_n	M_n
	**T**	**L_{13}**	**L_{14}**	**D**			
**R14**	0.31	0.15	0.26	0.28	0.58	120	8900
**R17**	0.30	0.14	0.26	0.28	0.58	147	11000

Supplementary material

^{1}H NMR spectrum of tinidazole

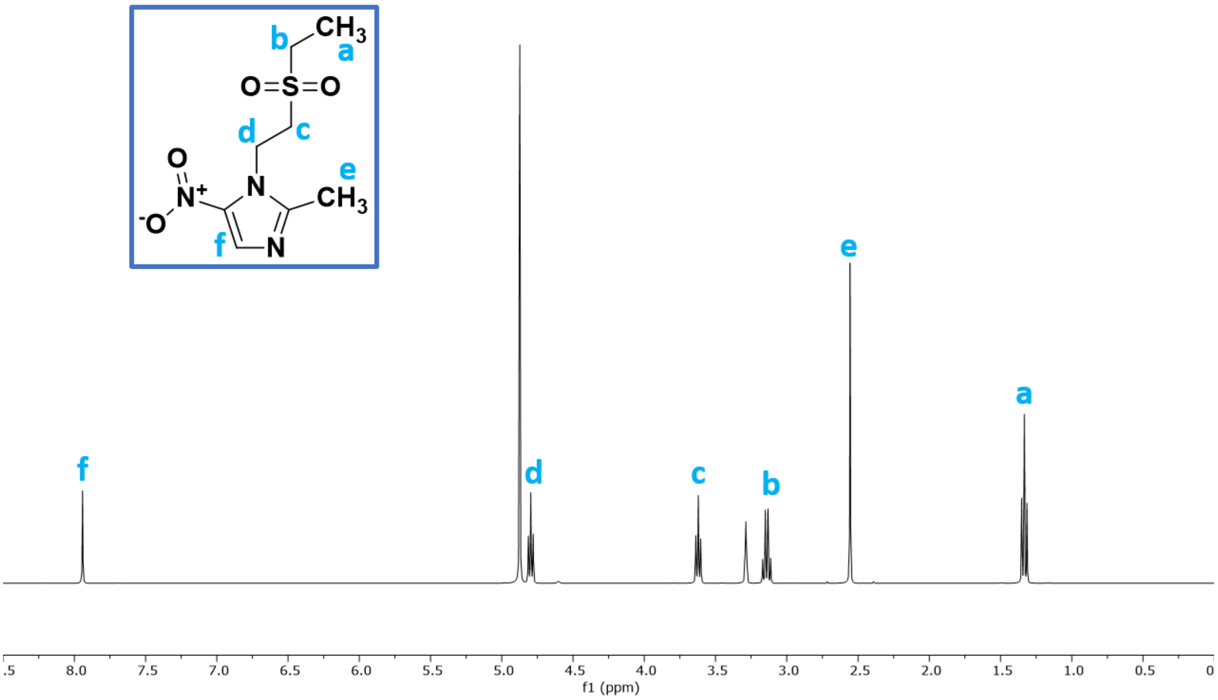

Figure S15. ^{1}H NMR spectrum of tinidazole recorded in MeOD

Supplementary material

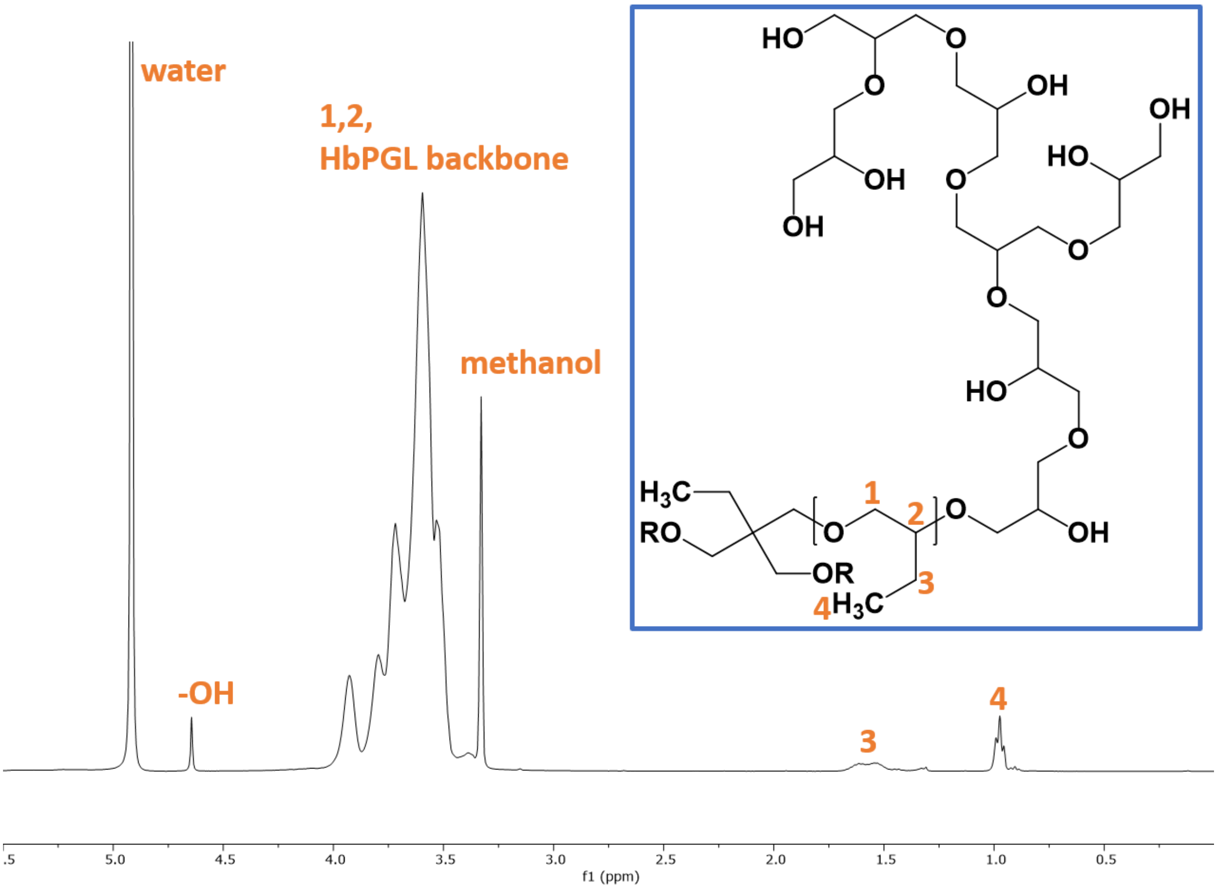

Figure S16. ^{1}H NMR spectrum of poly(1,2-epoxybutane)-*co*-HbPGL, R14 recorded in MeOD.

Supplementary material

Encapsulation tinidazole by R14

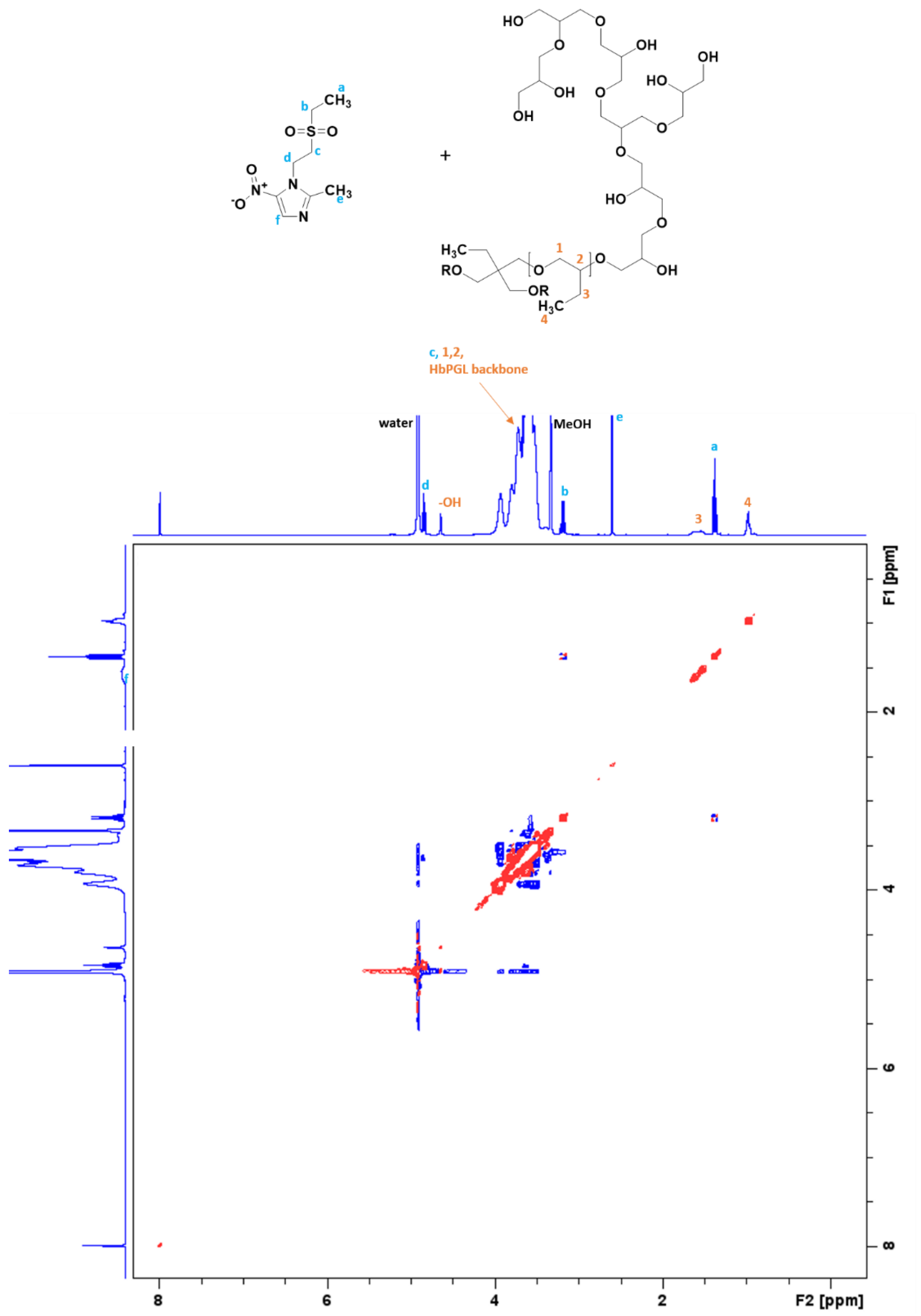

Figure S17: ^{1}H-^{1}H ROESY NMR spectrum of poly(1,2-epoxybutane)-*co*-HbPGL with tinidazole in MeOD. Drug to polymer ratio 15:1.

Supplementary material

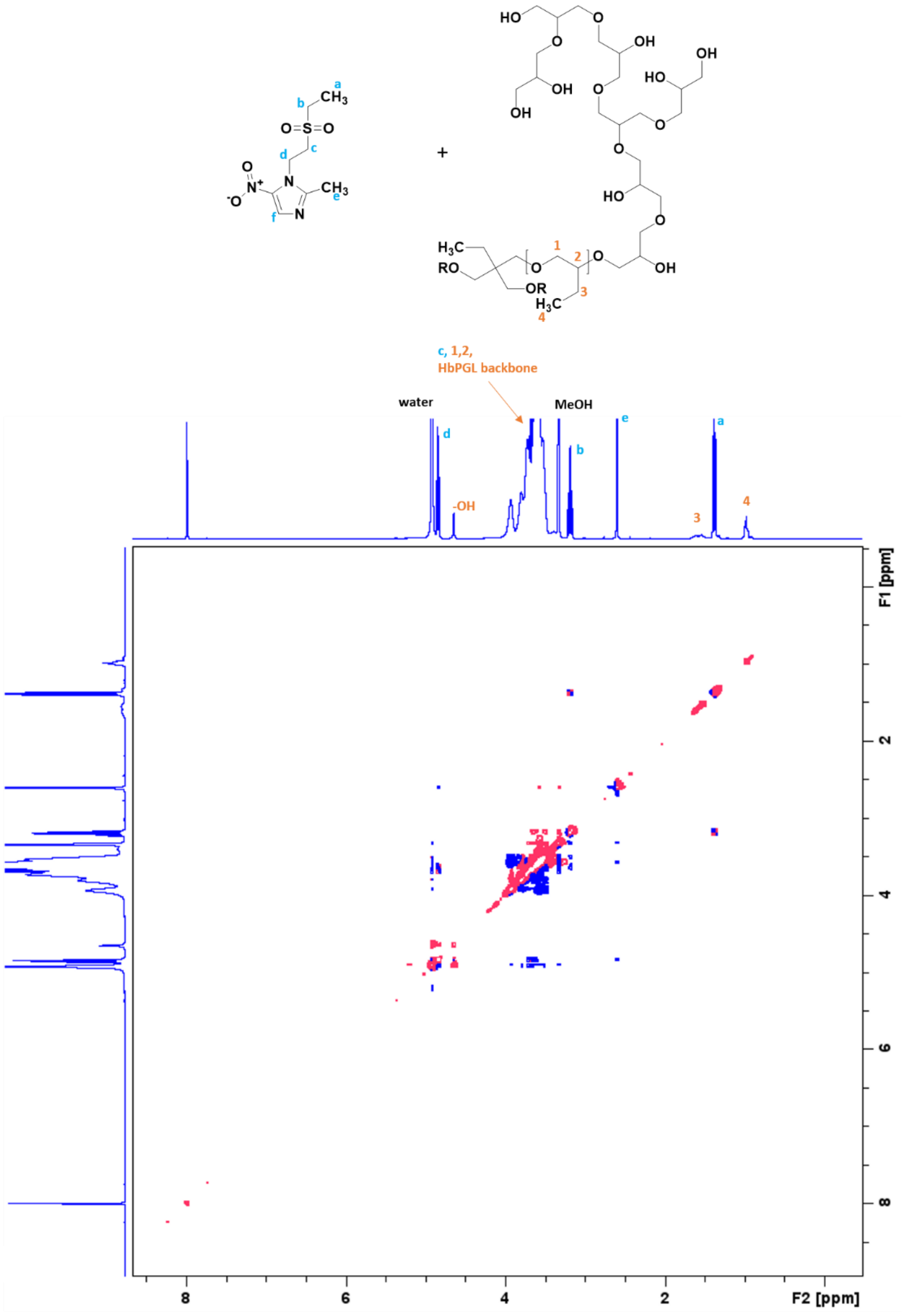

Figure S18: ^{1}H-^{1}H ROESY NMR spectrum of poly(1,2-epoxybutane)-*co*-HbPGL with tinidazole in MeOD. Drug to polymer ratio 45:1.

Supplementary material

Encapsulation tinidazole by R17

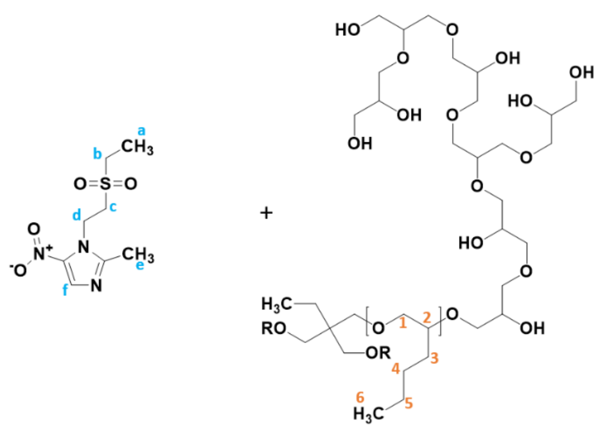

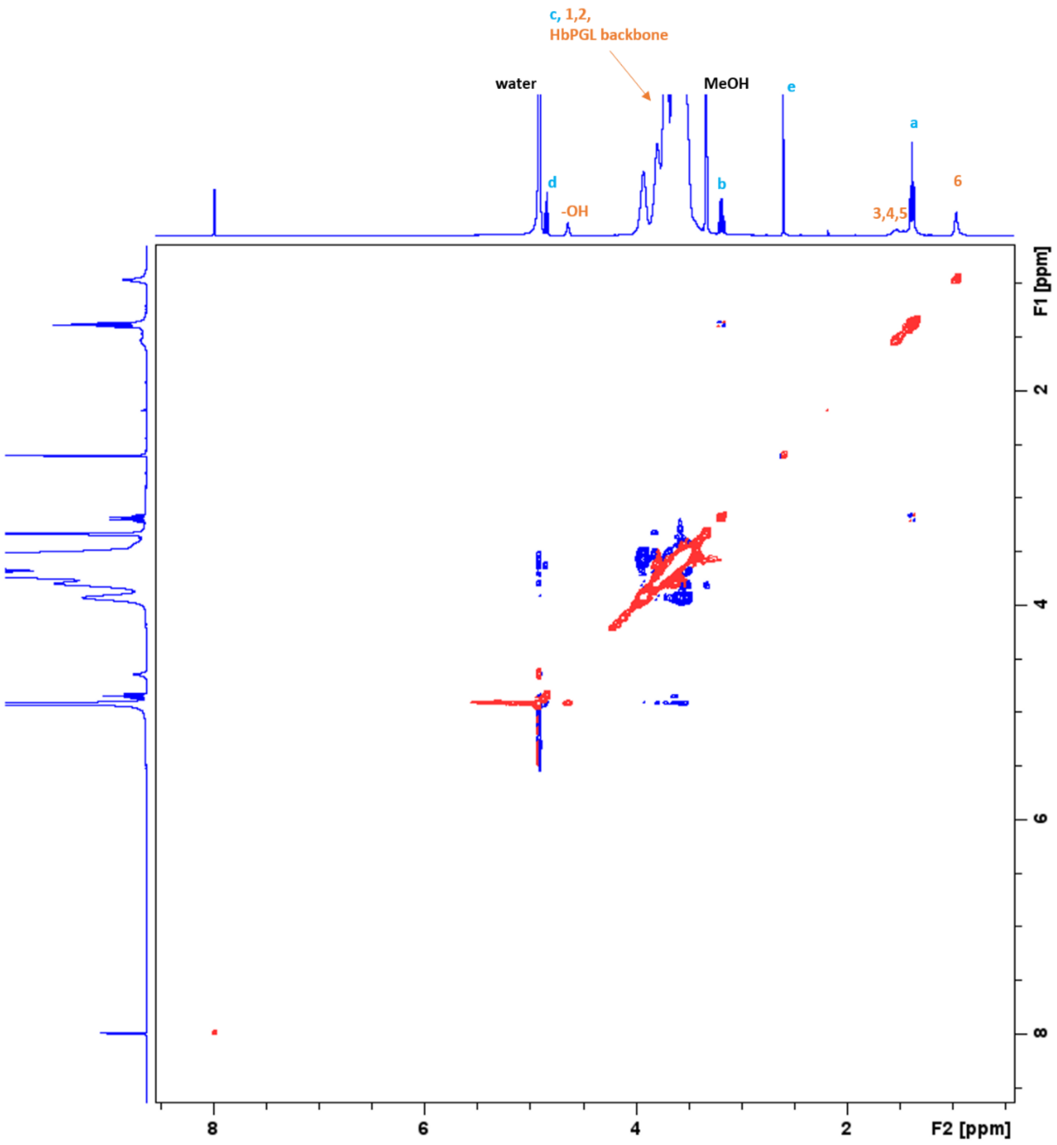

Figure S19: ^{1}H-^{1}H ROESY NMR spectrum of poly(1,2-epoxyhexane)-*co*-HbPGL with tinidazole in MeOD. Drug to polymer ratio 15:1.

Supplementary material

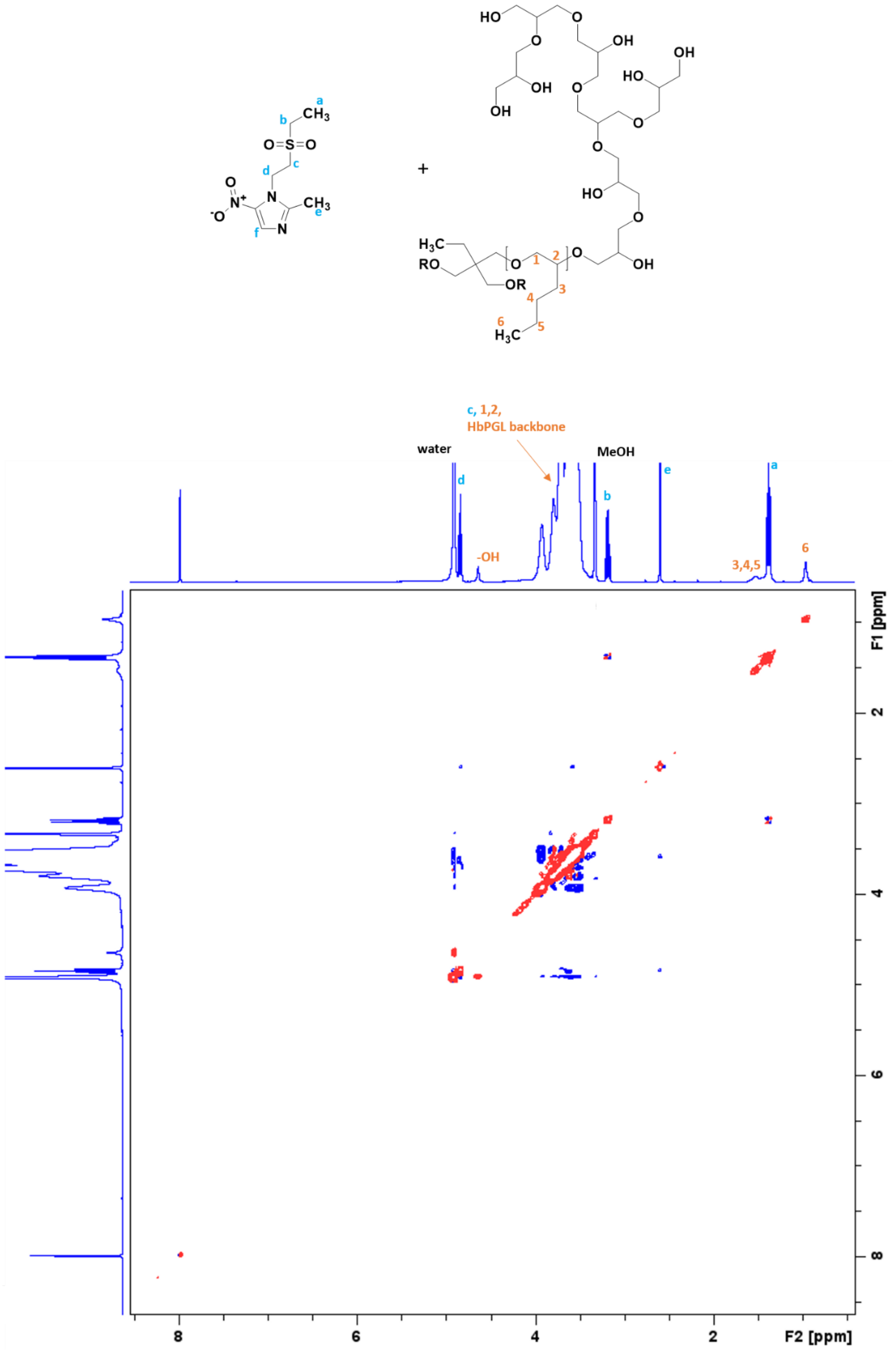

Figure S120: ^{1}H-^{1}H ROESY NMR spectrum of poly(1,2-epoxyhexane)-*co*-HbPGL with tinidazole in MeOD. Drug to polymer ratio 25:1.

Supplementary material

Encapsulation clotrimazole by R14

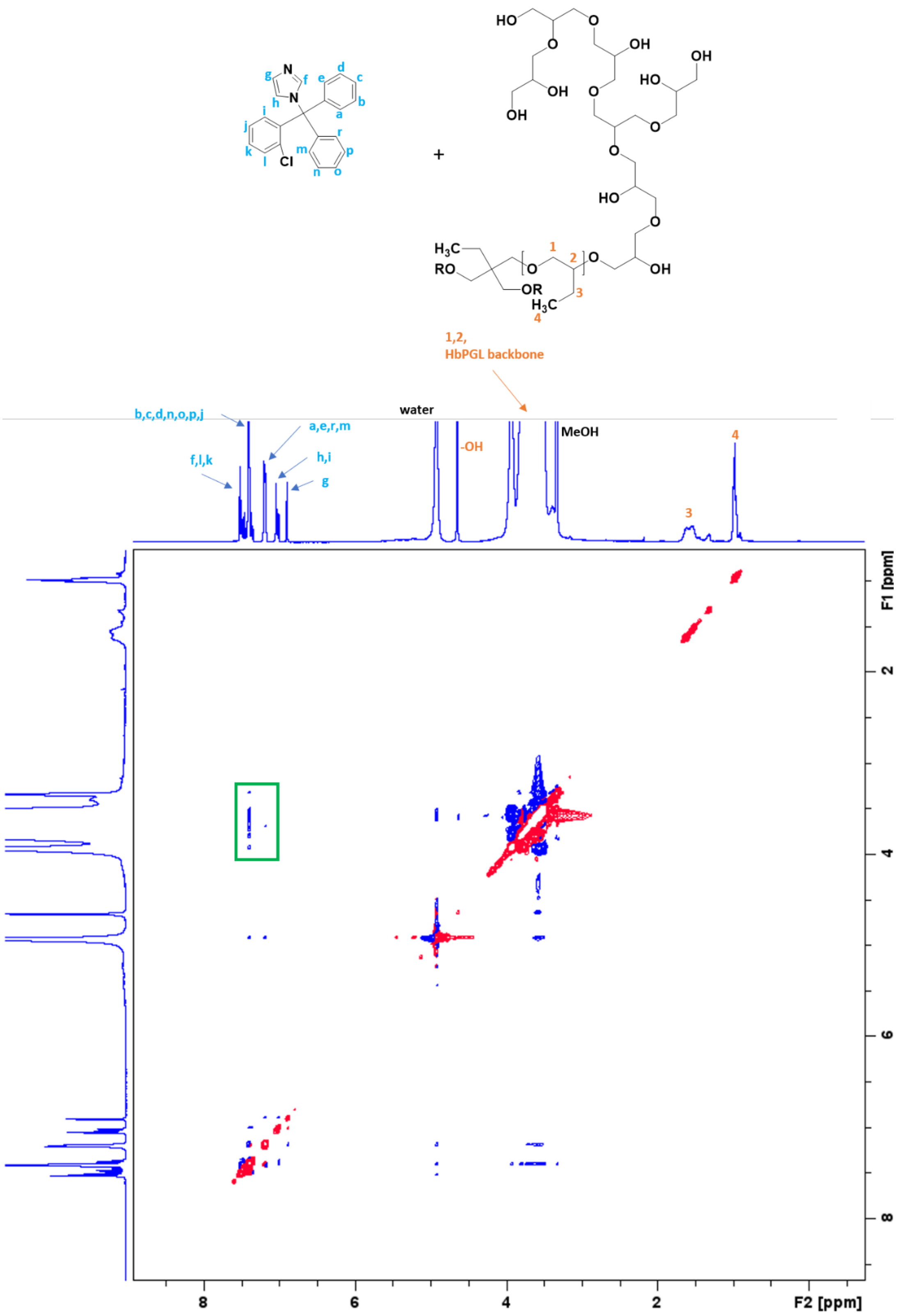

Figure S21: ^{1}H-^{1}H ROESY NMR spectrum of poly(1,2-epoxybutane)-*co*-HbPGL with clotrimazole in MeOD. Drug to polymer ratio 8:1.

Supplementary material

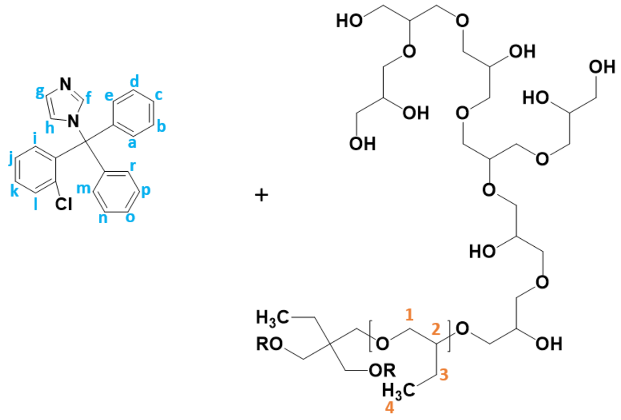

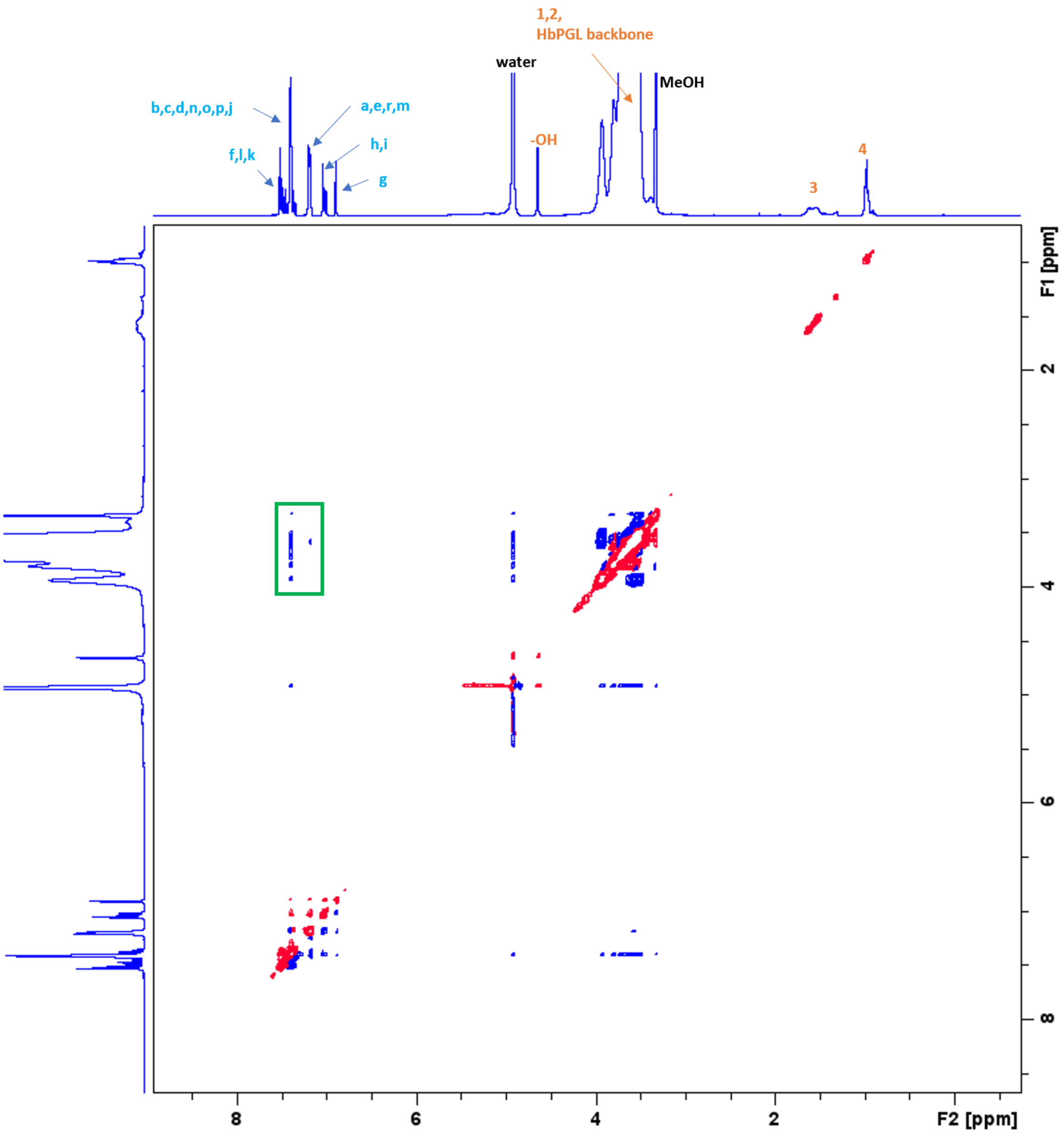

Figure S22: ^1H-^1H ROESY NMR spectrum of poly(1,2-epoxybutane)-*co*-HbPGL with clotrimazole in MeOD. Drug to polymer ratio 12:1.

Supplementary material

Encapsulation clotrimazole by R17

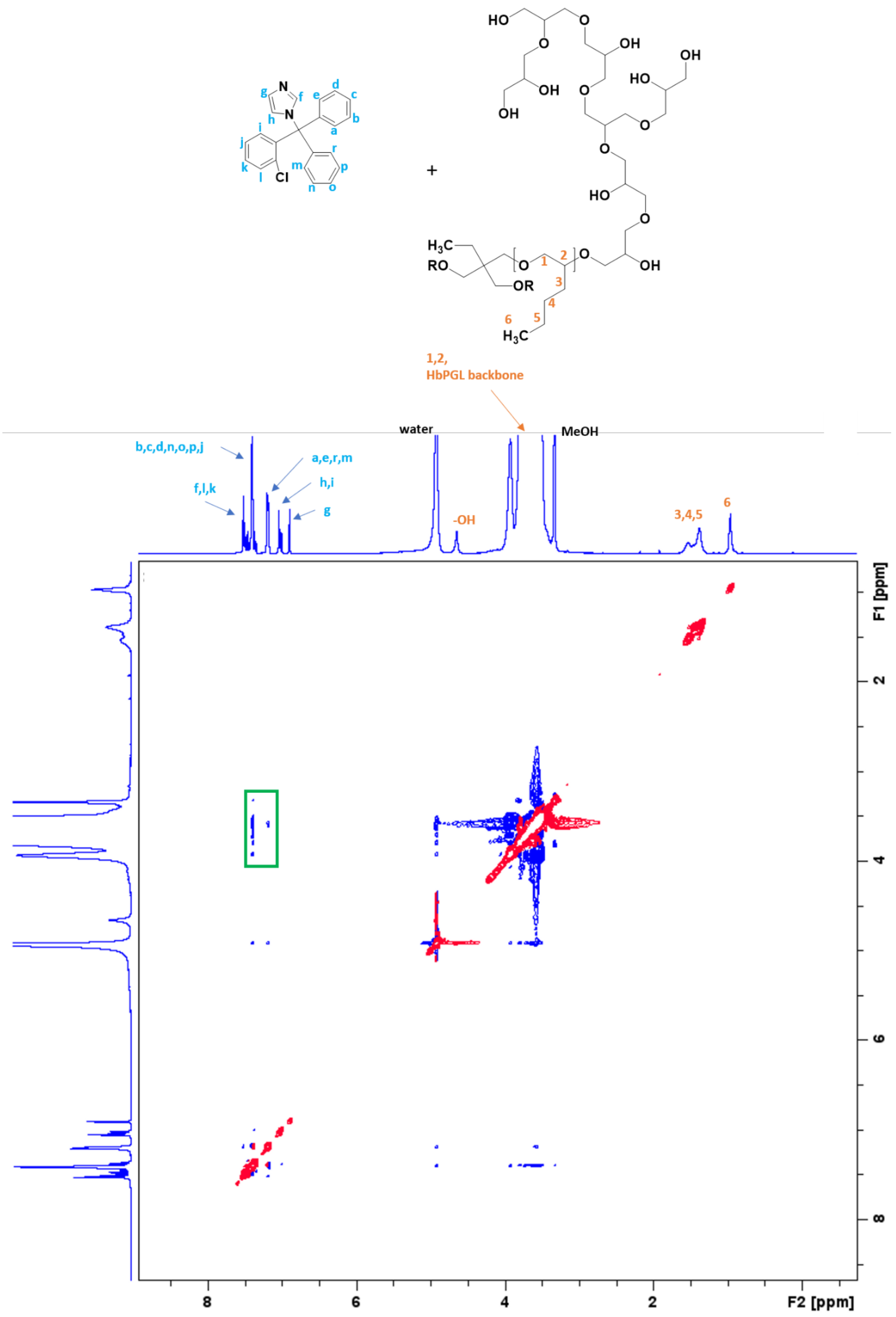

Figure S23: ^1H-^1H ROESY NMR spectrum of poly(1,2-epoxyhexane)-*co*-HbPGL with clotrimazole in MeOD. Drug to polymer ratio 12:1.

Supplementary material

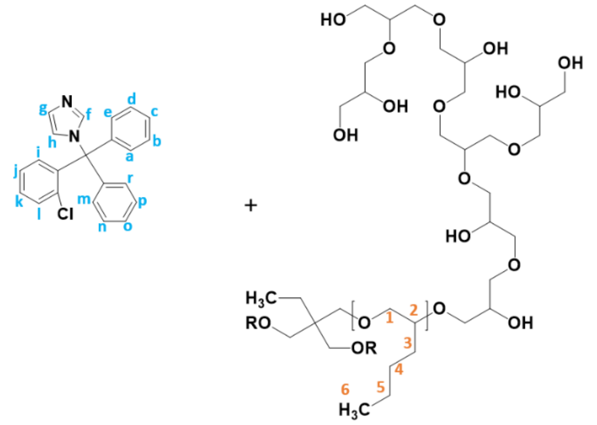

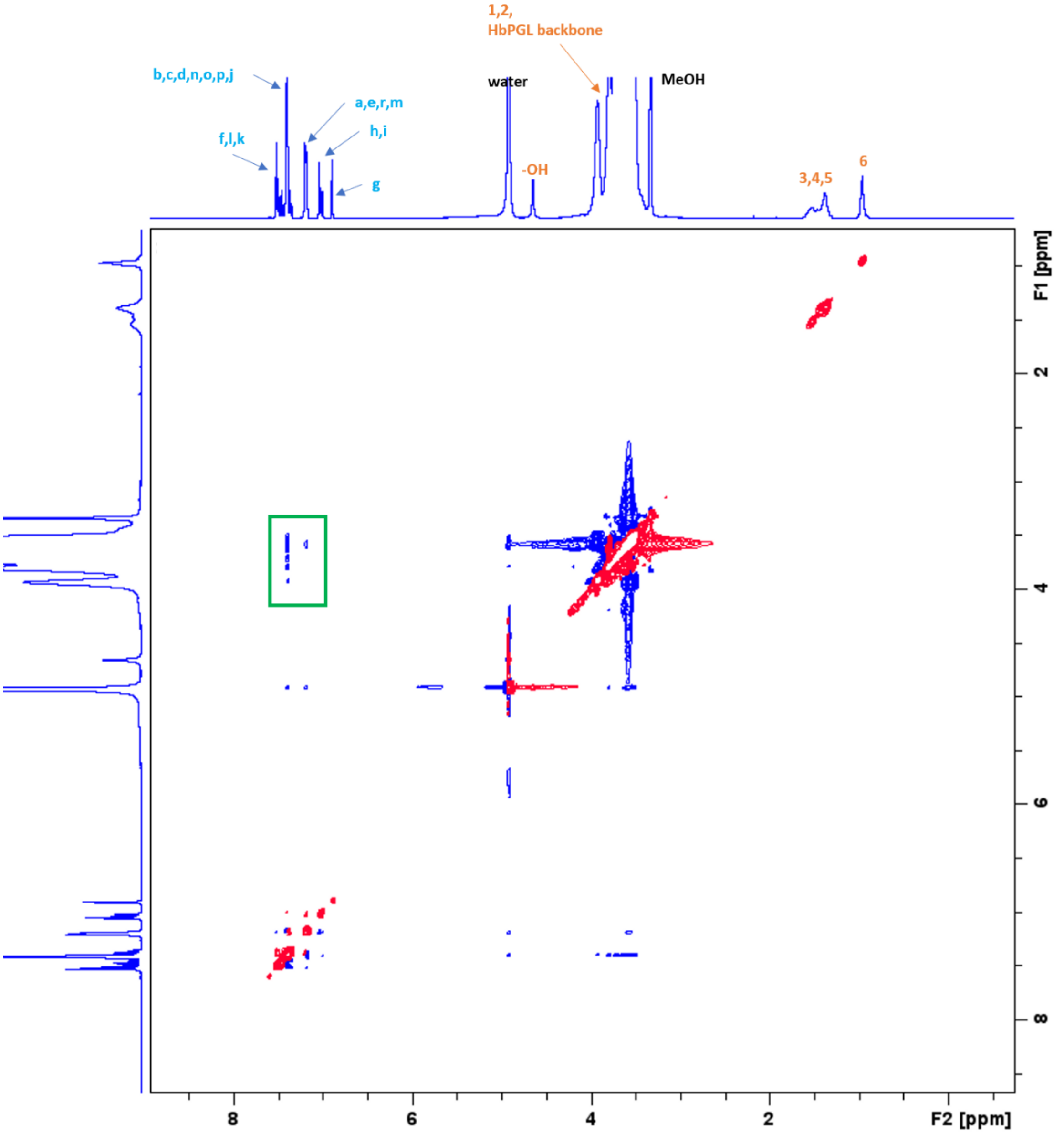

Figure S24: ¹H-¹H ROESY NMR spectrum of poly(1,2-epoxyhexane)-*co*-HbPGL with clotrimazole in MeOD. Drug to polymer ratio 15:1.

Supplementary material

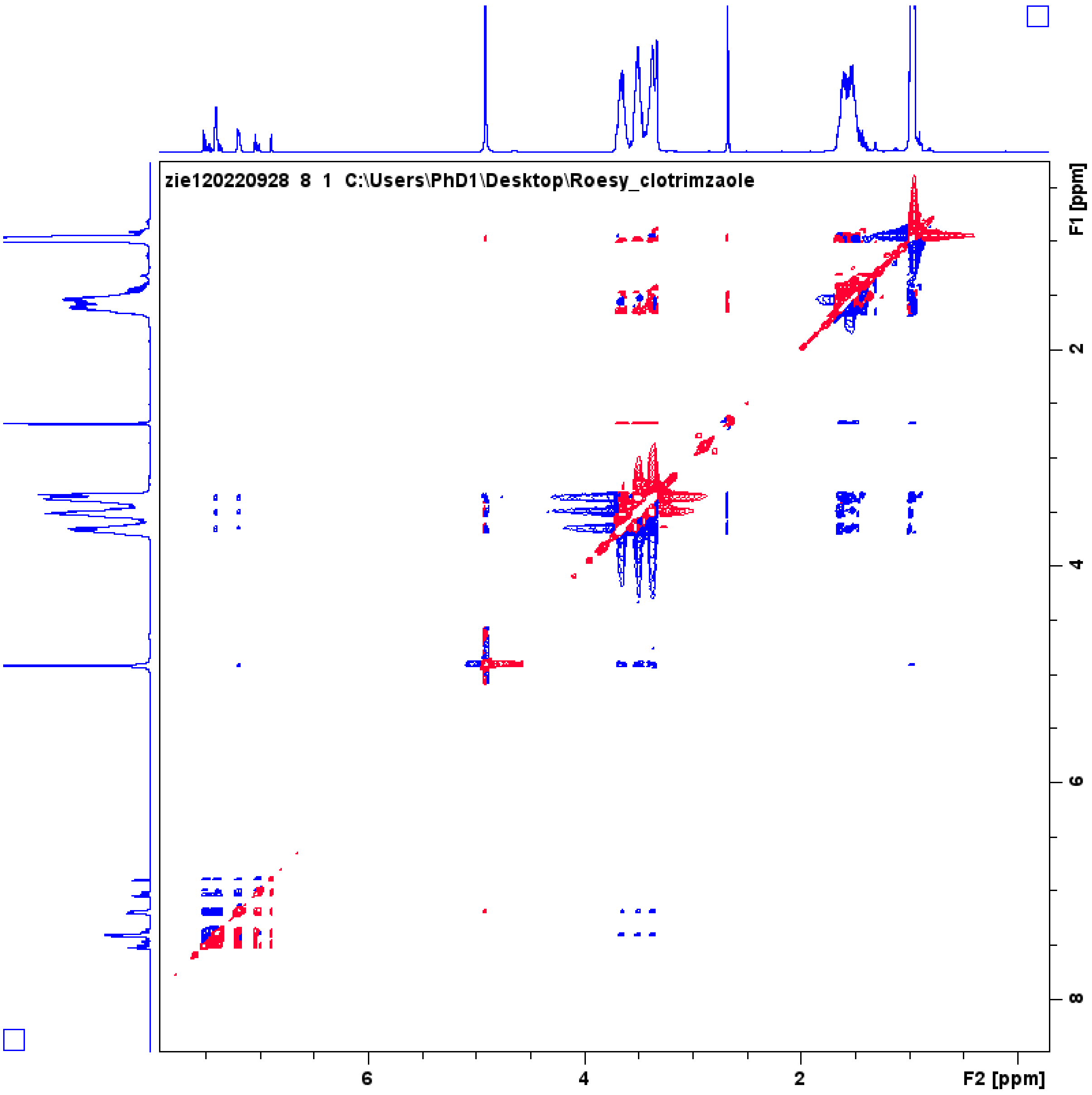

Figure S25: ^{1}H-^{1}H ROESY NMR spectrum of poly(1,2-epoxybutane)-*co*-HbPGL with clotrimazole.

Supplementary material

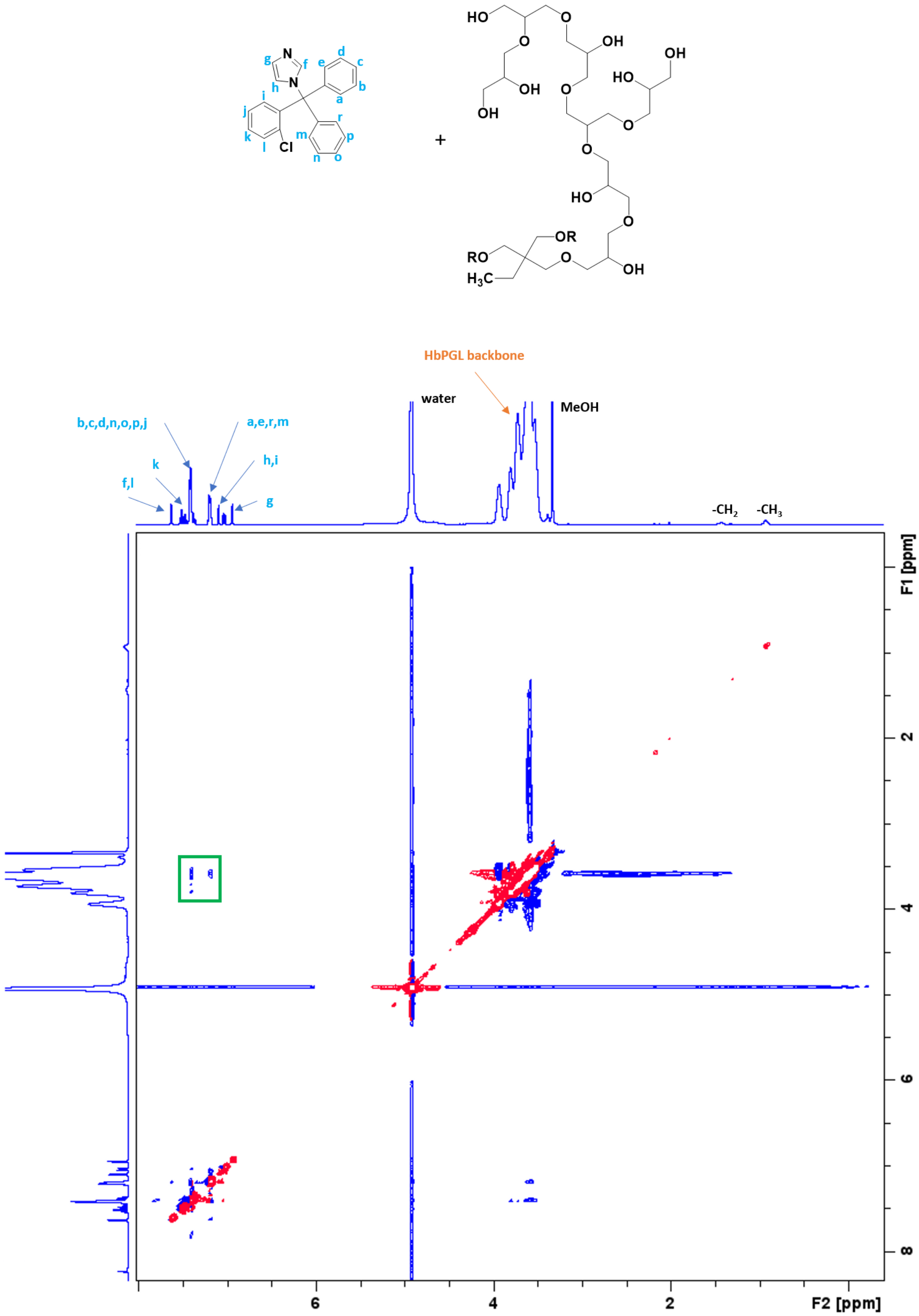

Figure S26: ^{1}H-^{1}H ROESY NMR spectrum of HbPGL with clotrimazole clotrimazole in MeOD.
